# IL-21 restricts T follicular regulatory T cell proliferation through Bcl-6 mediated inhibition of responsiveness to IL-2

**DOI:** 10.1038/ncomms14647

**Published:** 2017-03-17

**Authors:** Christoph Jandl, Sue M. Liu, Pablo F. Cañete, Joanna Warren, William E. Hughes, Alexis Vogelzang, Kylie Webster, Maria E. Craig, Gulbu Uzel, Alexander Dent, Polina Stepensky, Bärbel Keller, Klaus Warnatz, Jonathan Sprent, Cecile King

**Affiliations:** 1 Department of Immunology, Garvan Institute of Medical Research, 384 Victoria Street, Darlinghurst, New South Wales 2010, Australia; 2 Department of Medicine, St Vincent's Clinical School, University of NSW, Sydney, New South Wales 2010, Australia; 3 Division of Immunology and Genetics, John Curtin School of Medical Research, The Australian National University, Canberra, Australian Capital Territory 2601, Australia; 4 Institute of Endocrinology and Diabetes, The Children's Hospital at Westmead, Sydney, Locked Bag 4001, Westmead, New South Wales 2145, Australia; 5 School of Women's and Children's Health, University of New South Wales, High Street, Randwick, Sydney, New South Wales 2031, Australia; 6 Laboratory of Clinical Infectious Diseases, National Institute of Allergy and Infectious Diseases, National Institutes of Health, Bethesda, Maryland 20892-9806, USA; 7 Department of Microbiology and Immunology, Indiana University School of Medicine, 635 Barnhill Drive, MS 420, Indianapolis, Indiana 46202, USA; 8 Pediatric Hematology-Oncology and Bone Marrow Transplantation, Hadassah Hebrew University Hospital, Kiryat Hadassah, POB 12000, Jerusalem 91120, Israel; 9 Center for Chronic Immunodeficiency (CCI), University Medical Center and University of Freiburg, Breisacher Strasse 117, 79106 Freiburg, Germany

## Abstract

T follicular regulatory (Tfr) cells control the magnitude and specificity of the germinal centre reaction, but how regulation is contained to ensure generation of high-affinity antibody is unknown. Here we show that this balance is maintained by the reciprocal influence of interleukin (IL)-2 and IL-21. The number of IL-2-dependent FoxP3^+^ regulatory T cells is increased in the peripheral blood of human patients with loss-of-function mutations in the IL-21 receptor (IL-21R). In mice, IL-21:IL-21R interactions influence the phenotype of T follicular cells, reducing the expression of CXCR4 and inhibiting the expansion of Tfr cells after T-cell-dependent immunization. The negative effect of IL-21 on Tfr cells in mice is cell intrinsic and associated with decreased expression of the high affinity IL-2 receptor (CD25). Bcl-6, expressed in abundance in Tfr cells, inhibits CD25 expression and IL-21-mediated inhibition of CD25 is Bcl-6 dependent. These findings identify a mechanism by which IL-21 reinforces humoral immunity by restricting Tfr cell proliferation.

## Abstract (web summary)

IL-21 is central to follicular helper T cell function and germinal centre responses. Here the authors show that IL-21 signalling directly inhibits T follicular regulatory cells by limiting Bcl-6-dependent IL-2 receptor expression.

Cytokines provide cues that influence the growth, survival and differentiation of immune cells. The cytokines interleukin (IL)-2 and IL-21 are the products of neighbouring genes on chromosome 3 in mice and chromosome 4 in humans. The *Il2/Il21* locus has been associated with risk for several autoimmune and inflammatory diseases in genome-wide association studies^1^,^2^. *Il2* and *Il21* have similar intron and exon structures, suggesting that these two genes arose by gene duplication^3^,^4^. However, despite structural similarities, the gene products IL-2 and IL-21 are growth and differentiation factors for CD4^+^ T-cell subsets with distinct functions.

IL-2 is secreted by activated/effector T cells and is a survival factor for Forkhead Box P3 (Foxp3)-expressing regulatory T (Treg) cells, which are vital for regulating immune responses in mice^5^,^6^,^7^. In humans, a severe autoimmune disease immunodysregulation polyendocrinopathy enteropathy X-linked syndrome results from inactivating mutations in *FOXP3*, leading to dysfunction or deficiency in Treg cells^8^. The utilization of IL-2 by Treg cells is facilitated by binding of IL-2 by the cell surface expressed α-chain of the heterotrimeric high-affinity IL-2 receptor, comprising the IL-2Rα chain (CD25), the IL-2Rβ chain (CD122) and the common γ-chain (CD132)^9^.

The production of IL-21, in turn, is mostly restricted to T follicular helper (Tfh) cells that provide help to B cells for the generation of affinity-matured antibody during the germinal centre (GC) reaction^10^,^11^. The receptor for IL-21 is closely related to the IL-2Rβ^3^,^12^ and is broadly expressed on haematopoietic cells, where IL-21 imparts both autocrine and paracrine effects on lymphocytes that influence survival and differentiation^13^,^14^,^15^. In humans, loss-of-function mutations in *IL21* or *IL21R* cause a primary immunodeficiency syndrome associated with an increased susceptibility to chronic infections and gastrointestinal inflammation^16^,^17^,^18^,^19^. In addition to its roles in immunity, IL-21 contributes to the development of inflammatory and autoimmune diseases^13^.

Studies have revealed that IL-21-producing Tfh cells are controlled by a subset of IL-2-dependent FoxP3-expressing follicular Treg (Tfr) cells, a specialized subset of Foxp3^+^ Treg cells that co-localize during GC reactions within B-cell follicles^20^,^21^,^22^. FoxP3^+^ Tfr cells originate from natural (thymus-derived) Treg cells and acquire features of Tfh cells, such as expression of the B-follicular homing chemokine receptor CXCR5 (refs 20, 23) and high expression of the co-inhibitory molecule PD-1 (ref. 24). However, unlike Tfh cells, they lack expression of CD40L, IL-4 and IL-21 (refs 20, 21, 22). Tfr cells are suppressive and abrogating either Tfr cell development or their follicular localization enhances the GC reaction and antibody production^20^,^21^,^22^.

We have previously shown that Treg cells expand to a greater extent in *Il21*^*−/−*^ mice than in IL-21-sufficient mice after immunization and co-administration of anti-CD28 monoclonal antibodies^11^ and more recent studies have shown that IL-21:IL-21R signalling inhibits Treg expansion both *in vitro*^25^ and after lymphocytic choriomeningitis virus (LCMV) infection^26^. IL-21 has been shown to reduce FoxP3 expression, inhibit Treg suppressor function and influence Treg homeostasis by decreasing IL-2 production from effector T cells *in vitro*^25^,^27^,^28^,^29^,^30^,^31^. By contrast, IL-2 inhibits Tfh cell differentiation through activation of STAT5 and the subsequent repression of Bcl-6 (refs 32, 33, 34, 35).

In this study, we provide evidence that IL-21 has the capacity to reinforce the generation of a robust humoral immune response by inhibiting the proliferation of Tfr cells.

## Results

### IL-21 inhibits Treg cells

We have previously shown that Treg cell populations undergo greater expansion in *Il21*^*−/−*^ mice than in IL-21-sufficient mice following administration of anti-CD28 monoclonal antibodies in conjunction with immunization with the polyvalent antigen sheep red blood cells (SRBC)^11^. To further analyse the influence of IL-21 on Treg cells, we used intracellular immunostaining to distinguish total Foxp3^+^ CD4^+^ Treg and FoxP3^+^ Tfr cells in *Il21r*^−/−^ and wild-type (WT) mice following immunization with SRBC.

**Figure 1:**
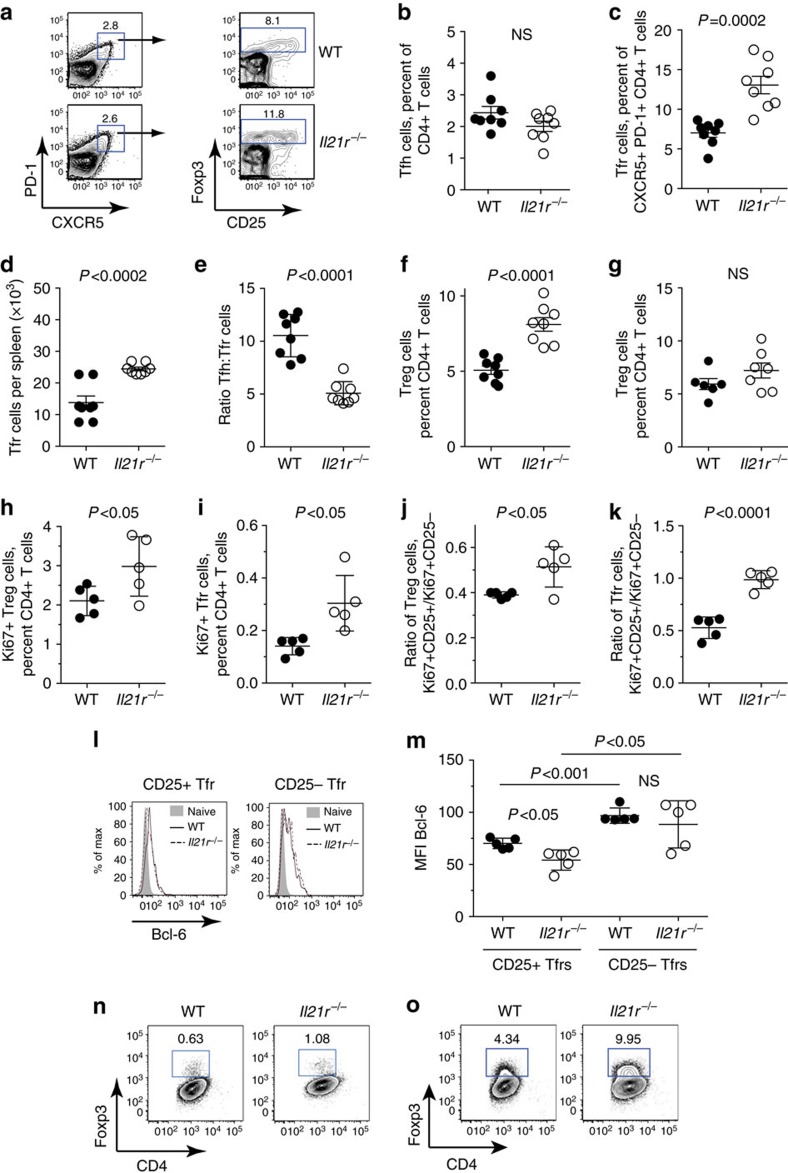
IL-21 inhibits the proliferation of Foxp3^+^ Treg cells. Eight-week-old WT *Il21r*^*+/+*^ and *Il21r*^*−/−*^ mice were immunized with 2 × 10^8^ SRBC intravenous and splenocytes were harvested on day 7 and stained for CD4, TCRβ, CXCR5, PD-1 and CD25 surface markers, and intracellular Foxp3 for flow cytometric analyses. (**a**) FACS dot plot shows gating strategy of CD4^+^ T cells for CXCR5^+^ PD-1^+^ Tfh cells and Foxp3^+^ CXCR5^+^ PD-1^+^ Tfr cells. (**b**) Percentage of Tfh cells within the CD4^+^ T-cell population, (**c**) percentage of Foxp3^+^ Tfr cells within the CXCR5^+^ PD-1^+^ CD4^+^ T follicular population, (**d**) absolute numbers of Tfr cells, (**e**) the ratio of Tfh cells to Tfr cells calculated per mouse and the percentages of Foxp3^+^ CD4^+^ Treg cells in (**f**) SRBC immunized and (**g**) unimmunized mice. Values shown from individual mice, including means ±s.d. from three replicate experiments where *n*=6–8 per group. Percentage of proliferative cells in *Il21r*^*−/−*^ and WT mice on day 7 following SRBC immunization as determined by immunostaining for Ki67: (**h**) Percentages of Ki67^+^ Treg cells. (**i**) Percentages of Ki67^+^ Tfr cells. (**j**) Ratio of CD25^+^:CD25^−^ cells within the Ki67^+^ Treg populations. (**k**) Ratio of CD25^+^:CD25^−^ cells within the Ki67^+^ Tfr populations. (**l**) Representative FACS histogram overlays of Bcl-6 immunostaining in CD25^+^ and CD25^−^ Tfr populations and (**m**) quantification of the mean fluorescence intensity of Bcl-6 immunostaining in CD25^+^ and CD25^−^ Tfr cells from *Il21r*^*−/−*^ and WT mice 7 days after SRBC immunization. Values shown from individual mice, including means ±s.d. from two replicate experiments. Statistical analyses were performed by unpaired Student's *t*-test, *P*-values >0.05 were deemed not significant (NS). Treg cell conversion efficiency was compared using splenic CD4^+^ T cells from WT and *Il21r*^*−/−*^ mice. T cells were cultured with plate-bound anti-CD3 and soluble anti-CD28 monoclonal antibodies (**n**) and with recombinant human transforming growth factor-β (TGFβ) (**o**) and Treg cell conversion was assessed on day 3. Contour plots show CD4^+^ 7-AAD^−^ cells and are representative of 4 independent experiments with similar results.

The percentages of Tfh cells, defined as CXCR5^hi^PD-1^hi^ CD4^+^ T cells, in the spleen were similar in *Il21r*^*−/−*^ and WT mice 7 days after immunization with SRBC (Fig. 1a,b and Supplementary Fig. 1). By contrast, *Il21r*^−/−^ Foxp3^+^ Tfr cells within the CXCR5^hi^PD-1^hi^ T follicular population were increased in the spleen compared with WT Tfr cells in terms of both the percentage (Fig. 1a,c) and absolute number of Tfr cells (Fig. 1d). The effect of IL-21R on the T follicular cell populations resulted in a significant (twofold) decrease in the Tfh:Tfr cell ratio (Fig. 1e). Tfr cells derive from Treg cells^20^ and both subsets were observed to express comparable amounts of the receptor for IL-21 (Supplementary Fig. 2). We similarly observed an increased fraction of *Il21r*^*−/−*^ total Foxp3^+^ Treg cells compared with WT Treg cells following SRBC immunization (Fig. 1f). In contrast to SRBC-immunized mice, the percentages of Treg cells in the spleen of unmaniplated *Il21r*^*−/−*^ and *Il21r*^*+/+*^ mice were similar (Fig. 1g). Thus, IL-21:IL-21R interactions, limit the expansion of both total Treg cells and Tfrs following immunization.

To determine whether the increased fraction of *Il21r*^*−/−*^ Tfr cells and Treg cells was associated with increased proliferation of these subsets, we immunostained for Ki67, which is a nuclear protein associated with cellular proliferation. A greater percentage of both *Il21r*^*−/−*^ Treg cells (Fig. 1h) and *Il21r*^*−/−*^ Tfr cells (Fig. 1i) were observed to express Ki67 than their WT counterparts on day 7 of SRBC immunization. The populations of FoxP3^+^ Treg cells and Tfr cells comprise both cells that express the high-affinity receptor for IL-2 (identified by expression of the α-chain of the IL-2R, CD25) and cells that lack CD25 (Fig. 1a). In this regard, it was of interest to note that the *Il21r*^*−/−*^ Ki67^+^ proliferating Treg population contained a greater fraction of CD25^+^ Treg cells relative to CD25^−^ Treg cells than the WT Ki67^+^ proliferating Treg population (Fig. 1j). This bias was more evident in the proliferating Tfr cell population, where CD25^+^ and CD25^−^ Ki67^+^ Tfr cells were present in equal ratios in *Il21r*^*−/−*^ mice, whereas in WT mice, CD25^+^ Tfr cells represented a minority of the Ki67^+^ population (Fig. 1k).

Previous studies have demonstrated that the expression of CXCR5 on Treg cells depends upon the transcriptional repressor Bcl-6 (ref. 21). Further analyses of the Tfr populations identified increased expression of Bcl-6 in Tfr cells that lack CD25 when compared with CD25^+^ Tfr cells (Fig. 1l,m). Furthermore, CD25^+^ Tfr cells from *Il21r*^*−/−*^ mice, despite exhibiting increased proliferation, exhibited lower expression of Bcl-6 than WT CD25^+^ Tfr cells (Fig. 1m). Collectively, these findings demonstrate that IL-21:IL-21R signalling reduces the proliferation of CD25-expressing Treg cells and Tfr cells, and indicate that responsiveness to IL-2 is associated with reduced expression of Bcl-6.

We next tested whether IL-21 had an influence on Treg expansion *in vitro*. In the presence of anti-CD3 and anti-CD28 monoclonal antibodies alone, *Il21r*^*−/−*^ CD4^+^ T-cell cultures exhibited a greater fraction of FoxP3^+^ Treg cells compared with WT CD4^+^ T-cell cultures (Fig. 1n and Supplementary Fig. 3). Moreover, *Il21r*^*−/−*^ CD4^+^ T cells cultured under polarizing (transforming growth factor-β) conditions exhibited an approximately twofold greater expansion of Foxp3^+^ Treg cells when compared with WT Treg cells cultured under the same conditions (Fig. 1o). Taken together, these findings indicate that IL-21 inhibits Treg expansion both in *vivo* and in *vitro*.

### Increased Foxp3^+^ T cells in IL-21R-deficient humans

In humans, loss-of-function mutations in the *IL21R* gene cause a primary immunodeficiency syndrome associated with recurrent and chronic infections^16^,^19^. To determine whether Treg cells were affected by IL-21R deficiency in humans, we analysed Treg cells and Tfr-phenotype cells in the blood of three patients P1 (B.II-1, a 13-year-old boy) and P2 (B.II-2, an 8-year-old boy), whose lymphocytes have previously been shown to exhibit a loss of IL-21-mediated signalling due to a homozygous deletion in exon 4 of the *IL21R* gene^16^, and P3 (a 5-month-old sister of a previously reported patient with the homozygous point mutation c.G602A)^19^. Treg cells in the peripheral blood of the IL-21R-deficient patients were compared with those in five age-matched healthy control subjects.

**Figure 2:**
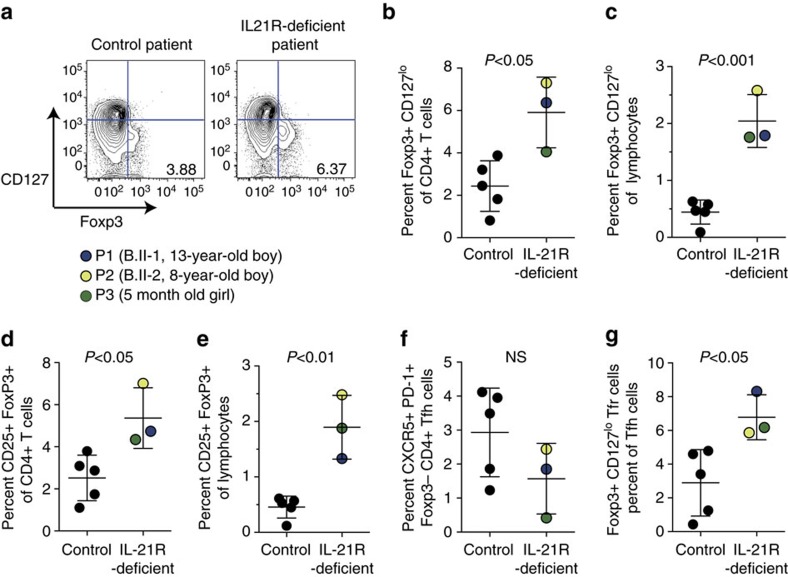
IL-21 limits Treg cells in humans. Analyses of CD4^+^ T cells in peripheral blood of human patients with loss of function mutation in *IL21R* compared with control patients. (**a**) Representative flow cytometry dot plots of peripheral blood from a healthy donor and an IL-21R-deficient patient (B.II-2) showing CD4^+^ CD3^+^ T cells immunostained for surface IL-7Rα (CD127) and nuclear Foxp3. Foxp3^+^ CD127^lo^ Treg cells are shown as percentage of CD4^+^ T cells (**b**) and as a percentage of lymphocytes (**c**) in three IL-21R-deficient patients compared with five healthy controls. FoxP3^+^ CD25^+^ Treg cells shown as a percentage of CD4^+^ T cells (**d**) and as a percentage of lymphocytes (**e**) in three IL-21R-deficient patients compared with five healthy controls. Data are shown for individual patients with means ±s.d. FoxP3^−^CXCR5^+^PD-1^+^ CD4^+^ Tfh cells (**f**) and Foxp3^+^CXCR^+^PD-1^+^ Tfr cells as a percentage of CXCR5^+^PD-1^+^CD4^+^CD3^+^ T cells (**g**) from peripheral blood of IL-21R-deficient patients and healthy controls shown as individual values with means ±s.d. *P-*values were determined by Student's *t*-test, *P*-values >0.05 were deemed not significant (NS).

Treg cells can be defined based on expression of CD4, CD25 and Foxp3. In humans, CD127 (IL-7Rα) is downregulated on Foxp3^+^ CD4^+^ T cells, including those that express medium levels of CD25 (refs 35, 36). Treg cells, identified as CD3^+^CD4^+^Foxp3^+^CD127^lo^ were markedly increased in the peripheral blood of all three IL-21R-deficient patients relative to healthy control patients, shown as a percentage of CD4^+^ T cells (Fig. 2a,b and Supplementary Fig. 4) and as a percentage of peripheral blood lymphocytes (Fig. 2c). Similarly, CD25^+^FoxP3^+^CD3^+^CD4^+^ Treg cells were increased within CD4^+^ T-cell populations (Fig. 2d) and total lymphocyte populations (Fig. 2e) of all three IL-21R-deficient patients compared with healthy controls.

FoxP3-expressing CD4^+^ T cells that exhibit a Tfr phenotype have previously been detected in the peripheral blood of mice^37^. Circulating and lymph node resident Tfr cells shared properties of memory cells, but blood Tfr cells were found to exhibit a relatively reduced suppressive capacity compared with lymphoid tissue-derived Tfr cells^38^. When we analysed follicular T-cell populations in the peripheral blood of the three IL-21R-deficient patients, we observed that the fractions of circulating CD4^+^CXCR5^hi^PD-1^hi^Foxp3^−^ Tfh phenotype cells were within the broad range of healthy controls (Fig. 2f), which is consistent with previous observations^19^,^39^. By contrast, the percentages of CD4^+^CXCR5^hi^PD-1^hi^CD127^lo^Foxp3^+^ Tfr phenotype cells in the peripheral blood of IL-21R-deficient patients were elevated relative to healthy controls (Fig. 2g). Thus, FoxP3^+^ total Treg cells and Tfr cells are increased in the blood of humans with loss-of-function mutations in the IL-21R and the expansion or increase in Treg cells occurs very early in life.

### IL-21:IL-21R signalling reduces CXCR4 on Tfh cells

**Figure 3:**
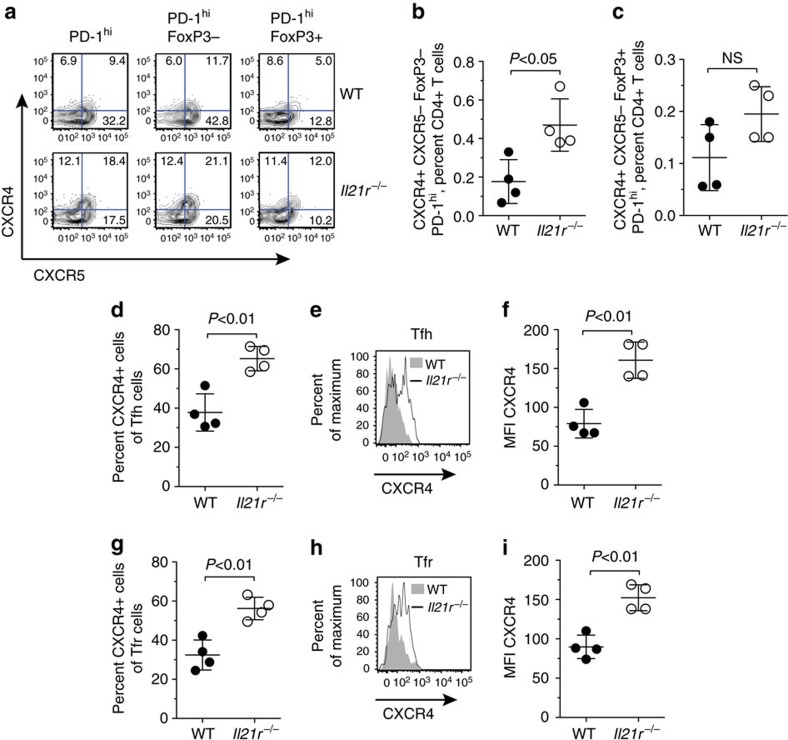
IL-21:IL-21R interactions reduce CXCR4 expression on Tfh cells. Eight-week-old WT *Il21r*^*+/+*^ and *Il21r*^*−/−*^ mice were immunized with 2 × 10^8^ SRBC (intravenous). On day 7, splenocytes were harvested and stained for CD4, TCRβ, CXCR5, PD-1 and CXCR4 surface markers and intracellular Foxp3. (**a**) Representative FACS dot plots showing expression of CXCR4 and CXCR5 on PD-1^hi^, PD-1^hi^ Foxp3^−^ and PD-1^hi^ Foxp3^+^ CD4^+^ T cells. (**b**) Percentages of CXCR4^+^ CXCR5^−^PD-1^hi^ Foxp3^−^ CD4^+^ extrafollicular T helper cells and (**c**) CXCR4^+^ CXCR5^−^PD-1^hi^ Foxp3^−^ CD4^+^ extrafollicular Treg cells in WT and *Il21r*^*−/−*^ mice. Percentages of (**d**) CXCR4 expressing Tfh cells (CXCR5^hi^ PD-1^hi^ FoxP3^−^ CD4^+^ T cells), (**e**) the mean fluorescence intensity (MFI) of CXCR4 expression on Tfh cells shown as a histogram overlay where WT Tfh cells are shown as a filled histogram and *Il21r*^*−/−*^ Tfh cells are shown as a black line, and (**f**) quantification of MFI of CXCR4 on WT and *Il21r*^*−/−*^ Tfh cells. Percentages of (**g**) CXCR4 expressing Tfr cells (CXCR5^hi^ PD-1^hi^ FoxP3^+^ CD4^+^ T cells) and the mean fluorescence intensity (MFI) of CXCR4 surface expression on WT and *Il21r*^*−/−*^ Tfr cells shown as (**h**) a histogram overlay and (**i**) quantification of MFI of CXCR4 on WT and *Il21r*^*−/−*^ Tfr cells. Data are shown from one representative experiment as individual mice and means ±s.d., from two experimental replicates with similar results. *P-*values were determined by Student's *t*-test, *P*-values >0.05 were deemed not significant (NS).

Tfh cells were observed in similar percentages in the presence and absence of IL-21R in humans and in mice following T-dependent immunization. Previous studies have shown that IL-6 can act redundantly to support Tfh cell differentiation in *Il21r*^*−/−*^ mice^40^,^41^. However, whether *Il21r*^*−/−*^ and WT Tfh cell populations are phenotypically distinct remains largely unexplored. C-X-C chemokine receptor type 4 (CXCR4) is a chemokine receptor expressed on T-extrafollicular helper cells^42^ and is also expressed on a fraction of Tfh cells^43^,^44^. Analyses of CXCR4^+^ PD-1^hi^ CD4^+^ Th cells on day 7 of SRBC-immunized *Il21r*^*−/−*^ and WT mice demonstrated that IL-21 significantly limits the expression of CXCR4 and the expansion of CXCR4^+^ PD-1^hi^ T cells (Fig. 3a). The percentages of *Il21r*^*−/−*^ CXCR4^+^ CXCR5^−^ PD-1^hi^ FoxP3^−^ Tfh cells were notably increased relative to WT extrafollicular T-helper cells (Fig. 3a,b). Interestingly, we also observed a fraction of previously undescribed CXCR4^+^ extrafollicular Treg cells (CXCR4^+^ CXCR5^−^ PD-1^hi^ FoxP3^+^ CD4^+^ T cells) in both *Il21r*^*−/−*^ and WT mice (Fig. 3a,c).

When we focused on Tfh cells, we observed that CXCR4 distinguished *Il21r*^*−/−*^ Tfh cells from WT Tfh cells. *Il21r*^*−/−*^ Tfh cells had an increased propensity to co-express CXCR4 (Fig. 3d) and IL-21:IL-21R interactions significantly reduced the expression of CXCR4 on Tfh cells (Fig. 3e,f). Interestingly, we observed a similar theme for Tfr cells 7 days after SRBC immunization, with increased percentages of CXCR4 expressing *Il21r*^*−/−*^ Tfr cells compared with WT Tfr cells (Fig. 3g). The intensity of CXCR4 expressed on the surface of *Il21r*^*−/−*^ Tfr was also significantly increased (Fig. 3h,i). These findings demonstrate that IL-21:IL-21R interactions influence the phenotype of follicular T-cell populations by restricting CXCR4-expressing Tfh and Tfr cells, and the intensity of expression of CXCR4.

In stark contrast to Th cells, CXCR4-expressing GC B cells were decreased in the absence of IL-21:IL-21R interactions. CXCR4-expressing GC B cells, a phenotype consistent with centroblasts^45^, were reduced in *Il21r*^*−/−*^ mice on day 7 following SRBC immunization and the intensity of CXCR4 expression was similarly decreased on *Il21r*^*−/−*^ GC B cells (Supplementary Fig. 5). Moreover, the reduced fraction of CXCR4 expressing cells and intensity of CXCR4 expression was specific to GC B cells, since the percentage of CXCR4^+^ cells and the intensity of CXCR4 were similar on the total *Il21r*^*−/−*^ and WT B cell populations (Supplementary Fig. 5).

### IL-21R intrinsic reduction of Tfr cells

The above findings indicated that IL-21 alters the phenotype of T follicular cells, but did not explain the marked expansion of Tfr cells in *Il21r*^*−/−*^ mice. To determine whether IL-21:IL-21R interactions had a cell intrinsic contribution to Treg cell subsets, we examined total Treg cells and Tfr cells during GC reactions that form following immunization in mixed bone marrow (BM) chimeras in mice consisting of WT BM expressing the congenic marker Thy1.1 and either *Il21r*^*−/−*^ Thy1.2 BM or control WT Thy1.2 BM delivered at 1:1 ratios into irradiated WT CD45.1 recipients. The host CD45.1 congenic marker enabled exclusion of host radioresistant T cells, which typically constitute 10% of the total T cells. Eight weeks later, and after checking chimerism following reconstitution, mice were immunized with SRBC and the relative contribution of WT and *Il21r*^*−/−*^ T cells to the percentages of follicular T cells and Treg cells were determined.

**Figure 4:**
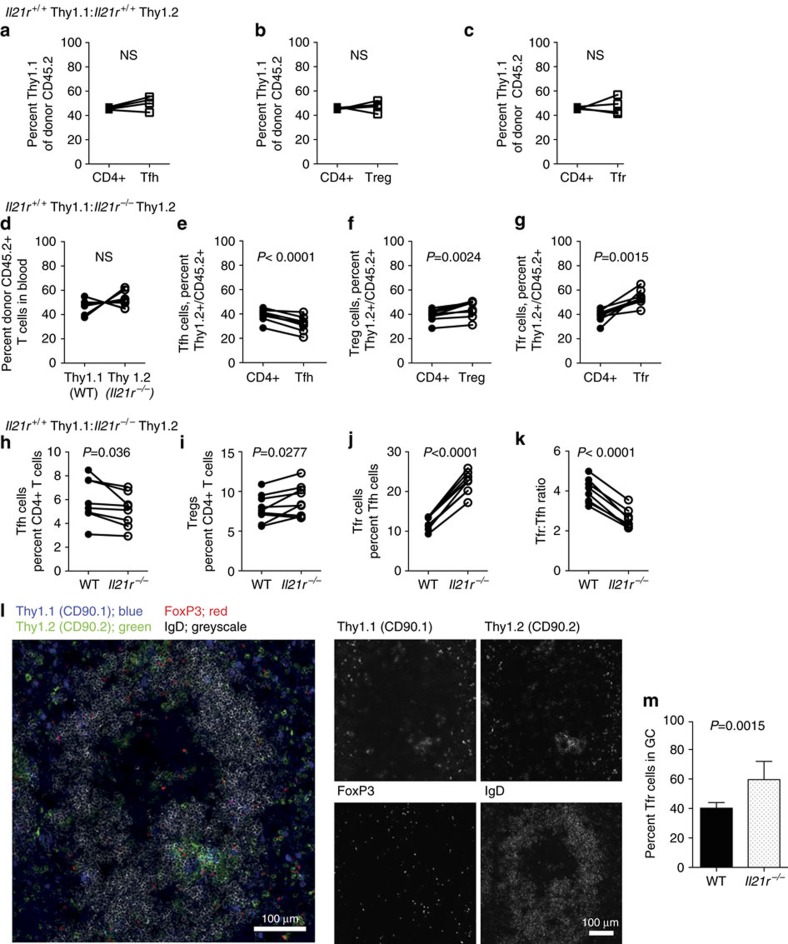
IL-21 has a cell intrinsic contribution to Tfr cell inhibition. Mixed BM chimeras were generated by reconstituting lethally irradiated 8-week-old CD45.1 recipients with 5 × 10^6^ each of CD45.2 BM cells from either *Il21r*^*+*^*/*^*+*^Thy1.1^+^ and *Il21r*^*−/−*^Thy1.2^+^ or control chimeras consisting of BM from *Il21r*^*+*^*/*^*+*^Thy1.1^+^ and *Il21r*^*+*^*/*^*+*^ Thy1.2 mice. Eight weeks after BM transfer, mice were immunized intravenously with 2 × 10^8^ SRBC and the CD4^+^ T-cell populations in the spleen were assessed on day 7. (**a**) Control chimeras showing relative percentages of Thy1.1 and Thy1.2 CD4^+^ T cells within the total CD45.2 CD4^+^ T cell population. (**b**) Relative percentages of Thy1.1 and Thy1.2 Foxp3^+^ Treg cells and (**c**) Thy1.1 and Thy1.2 Foxp3^+^CXCR5^+^PD-1^+^ Tfr cells within the donor CD45.2 populations, respectively. (**d**) Chimerism was assessed by tail bleed at 7 weeks. (**e**) Relative percentages of Thy1.2 *Il21r*^*−/−*^ CD4^+^ T cells within the total CD45.2 CD4^+^ T-cell population (shown in **e**–**g**) and Thy1.2 CXCR5^+^PD-1^+^ Foxp3^−^ Tfh cells. (**e**) Relative percentages of Thy1.2 Foxp3^+^ Treg cells. (**f**) Relative percentages of Thy1.2 Foxp3^+^ Treg cells and (**g**) relative percentages Thy1.2 Foxp3^+^ CXCR5^+^ PD-1^+^ CD4^+^ Tfr cells within the total CD45.2 Tfr population. Percentages of (**h**) Tfh cells as a percentage of CD4^+^ T cells. (**i**) Treg cells as a percentage of CD4^+^ T cells. (**j**) Tfr cells as a percentage of CXCR5^+^ PD-1^+^ CD4^+^ T follicular cells and (**k**) the ratio of Tfr:Tfh in individual mice within the Thy1.1 *Il21r*^*+/+*^ and Thy1.2 *Il21r*^*−/−*^ CD4^+^ donor populations in chimeras. Data are shown as individual mice *n*=5 from two separate experiments with similar results. Statistical analyses were performed by paired Student's *t*-test, *P*-values >0.05 were deemed not significant. Localization of Thy1.1 (WT) *Il21r*^*+/+*^ and Thy1.2 *Il21r*^*−/−*^ Foxp3^+^ cells within GCs of the spleen from mixed BM chimeras: GCs were identified as IgD^lo^. (**l**) Tfr cells within the GCs were identified with Foxp3 (red), Thy1.1 (blue) and Thy1.2 (green). Individual antibody immunostains are shown in grey scale. Scale bars, 100 μm. (**m**) Quantification of the percentage of Tfr cells counted from 80 immunostained GCs in sections of spleen as in **k**. Data are shown as mean ±s.d., *n*=5 mice.

Control chimeras consisting of 50% WT Thy1.1 BM and 50% WT Thy1.2 BM exhibited similar contributions of each congenic marker to the total CD4^+^ T cell (Fig. 4a–c and Supplementary Fig. 6), Tfh cell (Fig. 4a), Treg (Fig. 4b) and Tfr cell (Fig. 4c) populations. For chimeras consisting of 50% WT Thy1.1 BM and 50% *Il21r*^*−/−*^ Thy1.2 BM, equal fractions (50%:50%) of Thy1.1 WT and Thy1.2 *Il21r*^*−/−*^ lymphocytes were detected in blood before immunization (Fig. 4d). However, following immunization with SRBC, ∼39% of CD4^+^ T cells in the spleen were derived from Thy1.2 *Il21r*^*−/−*^ (Fig. 4e). CXCR5^hi^PD-1^hi^Foxp3^−^CD4^+^TCRβ^+^ Tfh cells exhibited similar proportions as total CD4^+^ T cells, with a slightly decreased fraction of Thy1.2^+^ *Il21r*^*−/−*^ Tfh cells (mean of 34.8% of the total donor CD45.2^+^ Tfh cells) derived from the Thy1.2^+^ *Il21r*^*−/−*^ CD4^+^ T-cell donor population (mean of 39% of CD45.2 CD4^+^ T cells) in each mouse (Fig. 4e). Similarly, there was only a small cell intrinsic contribution of IL-21R expression on Treg cells, with a slightly greater increase in Thy1.2 *Il21r*^*−/−*^ Treg cells (44.3%) relative to Thy1.2 *Il21r*^*−/−*^ CD4^+^ T cells (39%) within the total CD45.2^+^ CD4^+^ T-cell donor population of each mouse (Fig. 3f). By contrast, ∼39% of *Il21r*^*−/−*^ CD4^+^ T cells contributed 54% of the total CD45.2^+^ Tfr cell population, whereas 61% of WT Thy1.1^+^CD4^+^ T cells contributed only 46% of the total Tfr cells, demonstrating an increased conversion of *Il21r*^*−/−*^ cells to the Tfr phenotype relative to WT cells (Fig. 4g).

Parallel analyses of the T-cell subsets within each donor population showed that the fractions of *Il21r*^*−/−*^ and WT Tfh cells were similar, with a minor decrease in *Il21r*^*−/−*^ Tfh cells compared with WT Tfh cells observed (Fig. 4h). Similarly, *Il21r*^*−/−*^ Treg cells were observed at slightly higher percentages compared with WT Treg cells (Fig. 4i). By contrast, there was a markedly greater percentage of *Il21r*^*−/−*^ Foxp3^+^ Tfr cells compared with WT Tfr cells (Fig. 4j). The increased fraction of *Il21r*^*−/−*^ Tfr cells resulted in a twofold decrease in the overall ratio of Tfh:Tfr *Il21r*^*−/−*^ cells compared with WT cells (Fig. 4k). Detection of Foxp3^+^ cells in histological sections of the spleen in mixed BM chimeras identified both Thy1.1 WT and Thy1.2 *Il21r*^*−/−*^ Tfr cells within the IgD^lo^ GC areas (Fig. 4l). Enumeration of Foxp3^+^ Thy1.1^+^ and Thy1.2^+^ GC cells revealed that *Il21r*^*−/−*^ Thy1.2^+^ Tfr cells comprising a significantly greater percentage of the total GC Foxp3^+^ population (Fig. 4m). Taken together, these findings demonstrated that the cell-intrinsic effect of IL-21R was conspicuous for Tfr cells, but not for total Treg cells.

### IL-21 reduces expression of CD25 and responsiveness to IL-2

**Figure 5:**
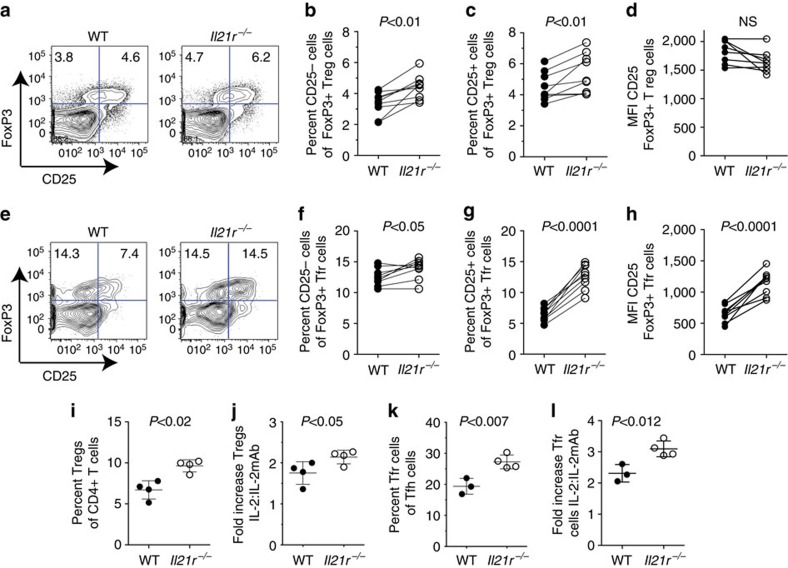
IL-21 limits responsiveness to IL-2. A direct comparison of CD25 expression on Thy1.1 *Il21r*^*+/+*^ cells and Thy1.2 *Il21r*^*−/−*^ Treg populations from mixed BM chimeras described in Fig. 4. (**a**) Representative FACS dot plot showing CD25 and Foxp3 expression in CD4^+^ T cells. Relative percentages of Thy1.1 *Il21r*^*+/+*^ cells and Thy1.2 *Il21r*^*−/−*^ cells in individual mice showing Foxp3^+^ CD4^+^ T cells that either (**b**) lack CD25 expression or (**c**) express CD25. (**d**) MFI of CD25 on Thy1.1 *Il21r*^*+/+*^ cells and Thy1.2 *Il21r*^*−/−*^ Foxp3^+^ CD4^+^ T cells. (**e**) Representative FACS dot plot showing CD25 and Foxp3 expression in CXCR5^+^ PD-1^+^ CD4^+^ T cells. Relative percentages of Thy1.1 *Il21r*^*+/+*^ cells and Thy1.2 *Il21r*^*−/−*^ Foxp3^+^ Tfr cells that either (**f**) lack CD25 expression or (**g**) express CD25. (**h**) MFI of CD25 on Thy1.1 *Il21r*^*+/+*^ cells and Thy1.2 *Il21r*^*−/−*^ Foxp3^+^ Tfr cells. IL-2:IL-2mAb complex expansion of Treg cells in *Il21r*^*−/−*^ and WT C57BL/6 mice: (**i**) Quantification of Foxp3^+^CD25^+^ Treg cells as a proportion of CD4^+^TCRβ^+^ T cells in IL2:IL2 monoclonal antibody-injected mice and (**j**) fold increase of Treg cells in response to IL2:IL2 monoclonal antibody treatment relative to SRBC immunized alone. (**k**) Quantification of Foxp3^+^ Tfr cells as a proportion of CXCR5^+^PD-1^+^CD4^+^TCRβ^+^ T cells and (**l**) fold increase of Tfr cells on IL2:IL2 monoclonal antibody treatment. Data are shown as individual mice with means ±s.d., from two replicate experiments, *P-*values were determined by Student's *t*-test, *P*-values >0.05 were deemed not significant (NS).

In addition to the analyses of the relative proportions of WT and *Il21r*^*−/−*^ CD4^+^ T-cell subsets in the SRBC-immunized mixed BM chimeras, we measured expression of IL-2Rα (CD25), which closely aligns with the Foxp3^+^ Treg population in mice. Treg cells in SRBC-immunized *Il21r*^*−/−*^:WT mixed BM chimeras demonstrated increased percentages of both CD25^−^ *Il21r*^*−/−*^ Treg cells (Fig. 5a,b) and CD25^+^ *Il21r*^*−/−*^ Treg cells (Fig. 5a,c) compared with WT Treg cells. However, equivalent high expression levels of CD25 were observed on WT and *Il21r*^*−/−*^ Foxp3-expressing (non-follicular) Treg cells (Fig. 5d). By contrast, we observed equivalent percentages of CD25^−^ Tfr cells derived from *Il21r*^*−/−*^ and WT BM (Fig. 5e,f), but the percentages of *Il21r*^*−/−*^ CD25^+^ Tfr cells were over twofold greater than WT CD25^+^ Tfr cells (Fig. 5e,g). These data indicate that within the same recipient mice, IL-21 modulated the expression of CD25 on Tfr cells, but not on the non-follicular Treg population. Furthermore, an expansion of *Il21r*^*−/−*^ Treg cells was observed in both the CD25^+^ and CD25^−^ populations, whereas the increased fraction of *Il21r*^*−/−*^ Tfr cells were predominantly those that expressed CD25.

Tfr cells are derived from Treg cells, but exhibited lower expression of CD25 (Fig. 5d,h). It was of interest to observe that in the absence of IL-21R, CD25 expression levels on Tfr cells approximated that of their Treg precursors (Fig. 5h). Given that Tfrs derive from Treg cells, this finding suggested that in mixed BM chimeras with presumably equivalent IL-2 bioavailability, the expression of CD25 on Tfr cells was reduced by IL-21 in a cell intrinsic manner. Taken together, these data show a more marked effect of IL-21 on Tfr cells, and that the relatively mild effect of IL-21 on CD25 expression on non-follicular Treg cells correlates with these cells already having maximum high CD25 expression. Therefore, our findings are consistent with an ability of IL-21:IL-21R signalling to limit responsiveness to IL-2.

As the expression of CD25 influences receptiveness to IL-2, we argued that *Il21r*^*−/−*^ Tfr cells should be more responsive to IL-2 than WT Tfr cells. To test this possibility, we administered IL-2:IL-2mAb complexes^46^ to SRBC-immunized *Il21r*^*−/−*^ and WT mice, to drive the expansion of CD25-expressing cells that occurs following SRBC immunization. A greater percentage of *Il21r*^*−/−*^ Treg cells than WT Treg cells was observed after IL-2:IL-2mAb administration (Fig. 5i). However, the fold increase in IL-2:IL-2mAb expanded Treg cells relative to Treg cells in control SRBC-immunized mice just reached significance (Fig. 5j). Consistent with the effect of the IL-2:IL-2mAb complexes on expansion of Treg cells, the percentage of *Il21r*^*−/−*^ Tfr cells was significantly greater than that of WT Tfr cells after IL-2:IL-2mAb treatment (Fig. 5k). However, in contrast to the Treg population, IL-2:IL-2mAb treatment induced a significantly greater expansion of *Il21r*^*−/−*^ Tfr cells, shown as an increase in the fold change above SRBC-immunized mice (Fig. 5l). Thus, Foxp3^+^CD4^+^ T cells expanded more efficiently in the absence of IL-21:IL-21R signalling and this was most pronounced in the Tfr population. The increased expansion in response to an excess of exogenous IL-2 by *Il21r*^*−/−*^ Tfr cells was associated with increased surface expression of CD25, the high-affinity IL-2 receptor. However, this relationship was not evident in the IL-2-dependent non-follicular Treg population, where CD25 was expressed similarly and at the highest levels on both WT and *Il21r*^*−/−*^ Treg cells.

A central function of STAT5 in T cells is to mediate signalling by IL-2. As there was no obvious difference in the level of expression of CD25 on the large non-follicular Treg population, we further tested whether the deficiency of IL-21R influenced Treg responsiveness to IL-2 by measurement of rmIL-2-induced phosphorylation of STAT5 by immunostaining and flow cytometry. Both WT and *Il21r*^*−/−*^ CD25^+^ Foxp3^+^ Treg cells exhibited increased STAT5 phosphorylation after 15 min of stimulation with rmIL-2 (Supplementary Fig. 7). However, *Il21r*^*−/−*^ cells exhibited increased pSTAT5 intensity and an increased percentage of Foxp3^+^ Treg cells containing pSTAT5 compared with WT cells in response to the same titration of rmIL-2 (Supplementary Fig. 2). Inclusion of exogenous rmIL-21 had no effect on rmIL-2-mediated phosphorylation of STAT5 in WT cells (Supplementary Fig. 2). This finding indicated that *Il21r*^*−/−*^ Treg cells were preconditioned for heightened signalling in response to IL-2.

### Bcl-6 inhibits CD25 expression on CD4^+^ T cells

We were intrigued that IL-21:IL-21R signalling had a more pronounced cell intrinsic effect on Tfr cells than Treg cells. As discussed earlier, Tfr cells derive from Treg precursors and upregulate Bcl-6 and CXCR5 (which is dependent upon Bcl-6)^47^, enabling their follicular localization. Therefore, we next determined whether IL-21 influenced CD25 expression directly and whether Bcl-6 mediated the effects of IL-21 on CD25 expression.

**Figure 6:**
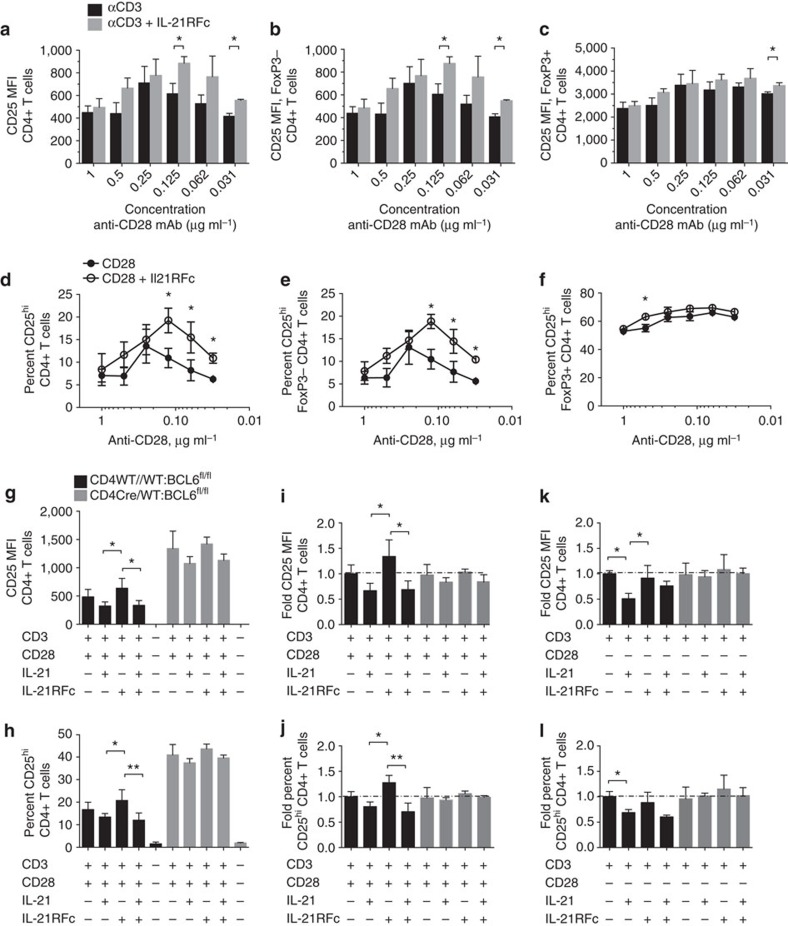
Bcl-6 inhibits CD25 and IL-21 modulates CD25 through Bcl-6. CD4^+^ T cells from the spleen of C57BL/6 mice were cultured *ex vivo* in the presence of anti-CD3 monoclonal antibody and decreasing concentrations of anti-CD28 monoclonal antibody, in the presence or absence of IL-21 neutralization (IL-21RFc) for 3 days. Bar graphs showing the immunofluorescence intensity (MFI) of the expression of CD25 on (**a**) TCRβ^+^CD4^+^ T cells (**b**) Foxp3^−^ TCRβ^+^CD4^+^ T cells and (**c**) Foxp3^+^ TCRβ^+^CD4^+^ T cells in the presence (grey bars) or absence (black bars) of 5 μg ml^−1^ of IL-21RFc. The percentages of CD25-expressing (**d**) TCRβ^+^CD4^+^ T cells, (**e**) Foxp3^−^ TCRβ^+^CD4^+^ T cells and (**f**) Foxp3^+^TCRβ^+^CD4^+^ T cells. Data are shown as the means ±s.d. of triplicate samples from a representative of two experiments with similar results. *P*-values were calculated by Student's *t*-test where *P<0.05. Bcl-6 inhibits CD25 expression on CD4^+^ T cells: CD4^+^ T cells were purified from mice in which Bcl-6 was conditionally deleted in CD4^+^ T cells (CD4Cre/WT:BCL6^fl/fl^) and Bcl-6-sufficient littermates (CD4WT/WT:BCL6^fl/fl^). Quantification of (**g**) the mean fluorescence intensity (MFI) of CD25 and (**h**) percentage of cells expressing high amounts of CD25 in Bcl6-sufficient (black bars) and Bcl6-deficient (grey bars) CD4^+^ T cells cultured in the presence of soluble anti-CD3 and anti-CD28 monoclonal antibodies and in the presence or absence of recombinant murine IL-21 and IL-21RFc. (**i**) Fold change of MFI relative to the mean value (dotted line) of CD4^+^ T cells stimulated with anti-CD3 and anti-CD28 monoclonal antibodies alone and (**j**) percentages of CD25-expressing CD4^+^ T cells. CD4^+^ T cells stimulated with anti-CD3 showing (**k**) fold change from the mean MFI (dotted line) and (**l**) percentages of CD25-expressing cells. Data are shown as the means ±s.d. of triplicate samples from a representative of two experiments with similar results. *P*-values were determined by one-way analysis of variance where significant differences between groups are shown as *P<0.05 and **P<0.01.

We first determined whether blockade of IL-21:IL-21R *in vitro* could mimic the increased expression of CD25 observed on *Il21r*^*−/−*^ CD4^+^ T cells. Culture of CD4^+^ T cells stimulated with soluble anti-CD3 monoclonal antibody and decreasing doses of soluble anti-CD28 monoclonal antibody over 3 days in the presence of IL-21-neutralizing IL-21RFc increased CD25 expression on both the total CD4^+^ T-cell population (Fig. 6a and Supplementary Fig. 7) and the Foxp3^−^ CD4^+^ T-cell population at lower doses of CD28 (<0.25 μg ml^−1^) (Fig. 6b and Supplementary Fig. 7). In contrast to the Foxp3^−^ CD4^+^ T-cell population, the intensity of CD25 expression on Foxp3^+^ Treg cells was not changed by IL-21 neutralization (Fig. 6c and Supplementary Fig. 7). The increased intensity of CD25 expression could be accounted for by an increased percentage of cells expressing high levels of CD25 on both the total CD4^+^ T-cell population (Fig. 6d) and the Foxp3^−^ population (Fig. 6e). By contrast, but consistent with the intensity of CD25 expression of Treg cells, the fraction of CD25-expressing Treg cells did not improve with the addition of IL-21RFc over the 3-day culture (Fig. 6f). Thus, neutralization of IL-21 improved responsiveness to IL-2 in CD4^+^ T cells and this was particularly evident under conditions of limiting co-stimulation by anti-CD28 monoclonal antibodies.

### IL-21 modulates CD25 expression via Bcl-6

IL-21 can upregulate the expression of Bcl-6 in CD4^+^ T cells^48^. To determine whether Bcl-6 mediated the effects of IL-21 on CD25 expression, we analysed the influence of IL-21 on CD25 expression on both CD4^+^ T cells and CD4^+^ T cells deficient in Bcl-6 *in vitro*. CD4^+^ T cells lacking Bcl-6 (CD4Cre/WT:BCL6^fl/fl^) exhibited increased expression of CD25 (Fig. 6g) and increased percentages of CD4^+^ T cells expressing high amounts of CD25 (Fig. 6h) in response to stimulation with anti-CD3 monoclonal antibody in the presence of anti-CD28 monoclonal antibody. There was a consistent trend of modulation of CD25 by IL-21 on WT (WT/WT:BCL6^fl/fl^) CD4^+^ T cells: when shown as fold change relative to the mean of CD3 monoclonal antibody and CD28 monoclonal antibody alone, the intensity of CD25 expression (Fig. 6i) and the percentages of CD25^hi^ CD4^+^ T cells (Fig. 6j) were reduced in the presence of exogenous rmIL-21 compared with IL-21RFc. However, IL-21RFc did not efficiently upregulate CD25 or increase CD25^hi^ CD4^+^ T cells in the presence of an excess of exogenous rmIL-21 (Fig. 6i,j, respectively). It was of interest to observe that IL-21 had a greater inhibitory effect on the intensity of CD25 expression and the percentages of CD25^hi^ CD4^+^ T cells in cultures stimulated with anti-CD3 monoclonal antibodies alone (Fig. 6k,l, respectively). Conversely, in the absence of CD28 costimulation, IL-21RFc neutralization was less effective at modulating CD25 expression (Fig. 6k,l). The ability of IL-21 to modulate CD25 expression was dependent upon expression of Bcl-6 in CD4^+^ T cells, as neither the intensity of CD25 on Bcl-6-deficient CD4^+^ (Cre/WT:BCL6^fl/fl^) T cells (Fig. 6g–i) nor the percentages of CD25^hi^ Bcl-6-deficient CD4^+^ T cells (Fig. 6j–l) were different either in the presence or absence of IL-21 or IL-21RFc. Taken together, these findings demonstrate that Bcl-6 inhibits CD25 expression on CD4^+^ T cells, and that IL-21 can limit the magnitude of CD25 expression and CD25-expressing CD4^+^ T cells through Bcl-6.

### IL-21 acts on Treg cells to modulate GC output

**Figure 7:**
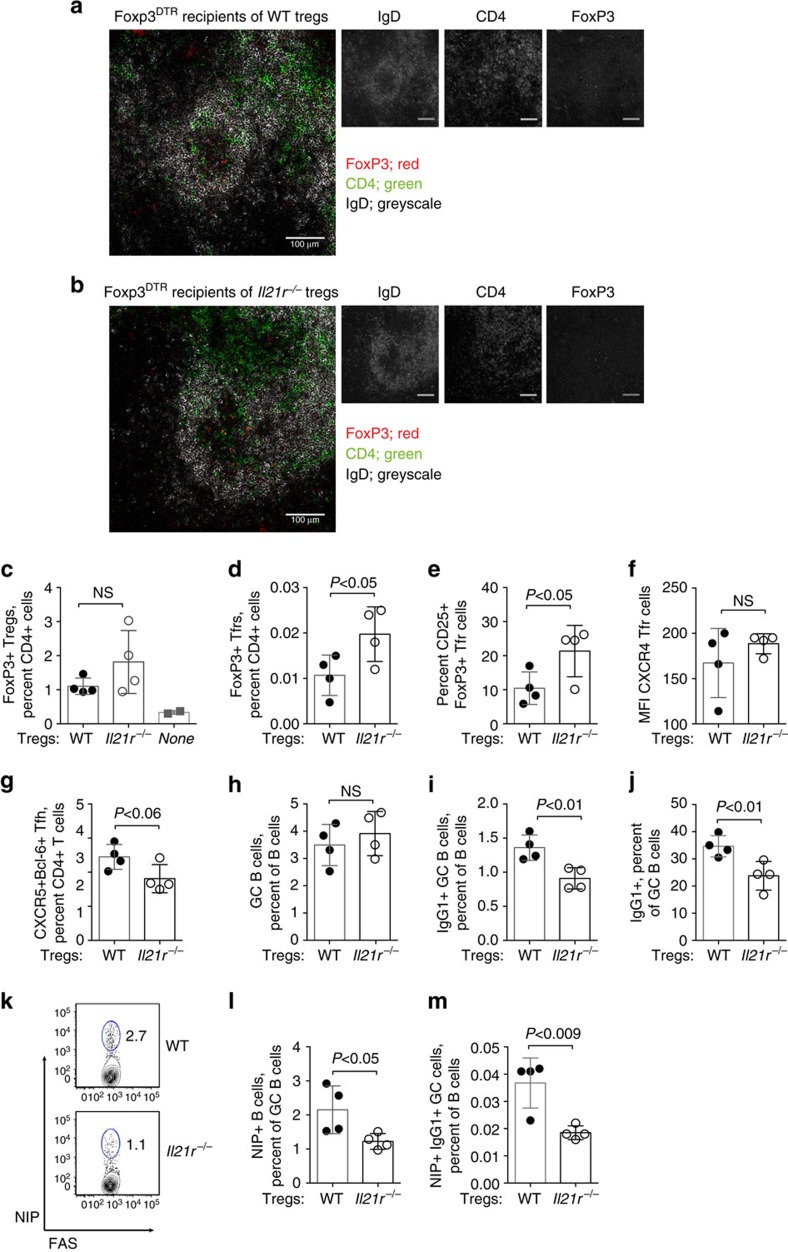
IL-21:IL-21R interactions on Treg cells reduce antigen-specific B-cell responses. FoxP3^DTR^ recipients depleted of FoxP3^+^ cells on days −1 and 4 of immunization received CD4^+^ Treg cells purified from the spleens of *Il21r*^*−/−*^ and WT mice before immunisation with NP-OVA adsorbed to alum. Localization of (**a**) FoxP3^+^ *Il21r*^*−/−*^ and (**b**) FoxP3^+^ WT Tfr cells within GCs of the spleens of Foxp3^DTR^ mice on day 10 of immunization. Histological sections of spleen were immunostained for IgD^+^ non-GC B cells (grey scale), FoxP3 (red) and CD4 (green). Individual antibody immunostains are shown in grey scale. Scale bars, 100 μm. Immunostaining and flow cytometric analyses on day 10 of NP-OVA immunization identified (**c**) FoxP3^+^ CD4^+^ Treg cells shown as a percentage of CD4^+^ T cells in Foxp3^DTR^ mice that received either WT, *Il21r^−/−^* or no FoxP3 Treg cells (**d**) FoxP3^+^ CXCR5^+^ PD-1^+^ CD4^+^ Tfr cells as a percentage of CD4^+^ T cells, (**e**) the percentage of CD25-expressing cells within the Tfr population, (**f**) the mean fluorescence intensity of CXCR4 immunostaining on Tfr cells and (**g**) CXCR5^hi^ Bcl-6^+^ Tfh cells. Analyses of the GC B-cell populations from the same mice showing the (**h**) percentage of FAS^+^ GL7^+^ GC B cells (**i**) IgG1^+^ GC B cells as percentage of B cells and (**j**) IgG1^+^ B cells as a percentage of the GC B cell population. (**k**) Flow cytometry dot plots showing gating for NP-specific FAS^+^ GC B cells, the (**l**) percentages of NP-specific (NIP^+^) GC B cells and (**m**) NP-specific IgG1^+^ GC B cells as a percentage of B cells. Data are shown as mean ±s.d., *n*=4 mice with two experimental replicates. *P-*values were determined by Student's *t*-test, *P*-values >0.05 were deemed not significant (NS).

To determine whether IL-21R expression on Treg cells had functional consequences in terms of GC output, we performed adoptive transfer of IL-21R-deficient (*Il21r*^*−/−*^) and IL-21R-sufficient (WT) Treg cells into Treg-deficient recipients. FoxP3^DTR^-recipient mice were treated with diphtheria toxin to remove endogenous Treg cells, before transfer of *Il21r*^*−/−*^ or WT Treg cells and immunization with 4-hydroxy-3-nitrophenyl acetyl-hapten (NP)-OVA adsorbed to alum. We immunized with NP-OVA to enable the identification of antigen (NP)-specific B cells. On day 10 of immunization, the spleens of recipient mice were analysed histologically showing localization of both *Il21r*^*−/−*^ Tfr cells (Fig. 7a) and WT Tfr cells (Fig. 7b) to the GC of FoxP3^DTR^ recipients.

Immunostaining and flow cytometric analyses on day 10 of NP-OVA immunization demonstrated similar percentages of Treg cells from *Il21r*^*−/−*^ and WT donors (Fig. 7c and Supplementary Fig. 7 for gating strategy), as well as efficient depletion of endogenous FoxP3^+^ CD4^+^ Treg cells by diphtheria toxin administration in mice that did not receive exogenous Treg cells (Fig. 7c). By contrast, Foxp3^DTR^ recipients of *Il21r*^*−/−*^ Treg cells contained a greater fraction of Tfr cells compared with recipients of WT Treg cells (Fig. 7d) and a greater fraction of *Il21r*^*−/−*^ Tfr cells expressed CD25 (Fig. 7e). CXCR4 was increased on Tfr cells in the absence of IL-21:IL-21R interactions (Fig. 4), but was not significantly different on Tfr cells from either donor genotype in FoxP3^DTR^ recipients (Fig. 7f). By contrast, the percentages of CXCR5^hi^ Bcl-6^hi^ Tfh cells were slightly decreased in recipients of *Il21r^−/−^* Treg cells (Fig. 7g). Thus, *Il21r*^*−/−*^ Tfr cells derived from adoptively transferred *Il21r*^*−/−*^ Treg cells and expand to a greater extent than WT Tfr cells, and this finding is consistent with our observations of the behaviour of *Il21r*^*−/−*^ Tfr cells in mixed BM chimeras (Fig. 4).

Analysis of the effect of *Il21r*^*−/−*^ and WT Treg transfer on the GC B-cell population showed equivalent percentages of GC B cells in FoxP3^DTR^ recipients of *Il21r*^*−/−*^ and WT Treg cells (Fig. 7h and Supplementary Fig. 8). However, a closer examination of the GC B-cell population revealed that recipients of *Il21r*^*−/−*^ Treg cells harboured reduced percentages of IgG1^+^ GC B cells (Fig. 7i), with a relative reduction of IgG1^+^ cells within the total GC B-cell population (Fig. 7j). Furthermore, FoxP3^DTR^ recipients of *Il21r*^*−/−*^ Treg cells had slightly reduced percentages of NP-specific GC B cells (Fig. 7k,l) and, in keeping with the reduced IgG1^+^ GC B cell population, reduced percentages of IgG1^+^ NP-specific GC B cells (Fig. 7m). These data suggest that the increased percentages of *Il21r*^*−/−*^ Tfr cells had the functional effect of reducing the percentages of IgG1^+^ GC B cells.

## Discussion

The discovery of Treg cells that function within the B-cell follicle provided evidence that regulation of antibody production occurs within the GC niche^20^,^21^,^22^. Our study provides insights into how the interplay between two neighboring genes, namely *Il2* and *Il21*, can shape antibody responses. IL-21 activates the Jak–STAT signalling pathway, leading to phosphorylation of STAT1 and STAT3, whereas IL-2 strongly activates STAT5. The ability of different STAT molecules to compete for STAT binding sites and affect gene transcription may underpin the relative influence of IL-2 and IL-21 on Tfh and regulatory subsets^31^,^32^,^33^,^34^,^49^. The relative influence of IL-2 and IL-21 on T follicular cells is likely to be dictated by several factors including the localization of cytokine production within the T-cell areas and B-cell follicles of secondary lymphoid organs^50^,^51^.

Both total FoxP3^+^ Treg cells and Tfr-phenotype cells were increased in the blood of IL-21R-deficient humans, suggesting that IL-21:IL-21R signalling has a negative influence on human Treg cells. CD25^hi^ CD4^+^ T cells in peripheral blood were previously shown to be similar between IL-21R-deficient patients and healthy controls^19^,^39^, and our findings are consistent with these observations. By contrast, dual expressing FoxP3^+^CD25^hi^ Treg cells were increased by IL21R deficiency. We also identified an increase in the fraction of Tfr-phenotype cells in peripheral blood, but the relationship of these cells with Tfr cells described in secondary lymphoid organs remains unknown. The initial study describing immune dysregulation in IL-21R defective patients reported that they exhibit recurrent and chronic infections^16^. Suppression of the immune response to infection by Treg cells in humans has been suggested previously. For instance, in HIV infection, the Foxp3^+^CD127^lo^ CD4^+^ T-cell population may be particularly important in limiting immune activation^52^. The increase in Treg cells and Tfr-phenotype cells in IL-21R-defective patients is a remarkable finding, but whether the expanded population of Treg cells contributes to the inability to control chronic infection in these patients remains unknown.

In mice, the cell-intrinsic effect of IL-21R was more conspicuous for the follicular subset of Foxp3^+^ T cells than for the broader Treg population. Previous studies have demonstrated that IL-21 has direct negative effects on Treg cells and can destabilize FoxP3 expression^27^,^28^,^29^,^30^,^53^. IL-21 can inhibit both Treg expansion following LCMV infection in a cell intrinsic manner^26^ and Foxp3^+^CXCR5^+^ICOS^+^CD4^+^ cells in autoimmune BXD2 mice^54^. In addition, another recent study demonstrated that IL-21 limits Treg cells in a cell extrinsic manner by inhibiting IL-2 production from conventional T cells *in vitro*^25^ and our findings are consistent with IL-2 or other extrinsic factors contributing to the size of the total Treg population. Here we describe a mechanism that may connect previous observations that the inhibition of Tfr cell expansion by IL-21 was associated with a decreased expression of CD25 and responsiveness to IL-2.

IL-21-mediated suppression of the α-chain of the high-affinity IL-2 receptor (CD25) was evident on Tfr cells following SRBC immunization, as *Il21r*^*−/−*^ Tfr cells expressed CD25 at the high or maximum levels observed on non-follicular Treg cells. The FoxP3^+^ Tfr population comprises both CD25^+^ and CD25^−^ cells, and we observed that the CD25^+^ Tfr population proliferated to a greater extent in the absence of IL-21:IL-21R interactions. IL-21 reduced and neutralization of IL-21 improved responsiveness to IL-2 in CD4^+^ T cells *in vitro* and IL-21 was most effective under conditions of limiting co-stimulation by anti-CD28 monoclonal antibodies. As one of the major functions of CD28 is to augment IL-2 production^55^, these findings are consistent with IL-21 being less efficient at limiting CD25 expression and Tfr cell expansion where IL-2 is abundant. Previous studies have demonstrated that *Cd25* is a target of Bcl-6, binding to an intron within *Cd25* (ref. 56). Our findings suggest that other cytokines that can upregulate Bcl-6 may also modulate CD25 expression on Tfr cells. In this regard, adoptively transferred TCR transgenic Smarta (SM) CD4^+^ T cells and SM Th1 cells retained a notably higher level of CD25 expression in *Il6*^*−/−*^ recipient mice compared with SM cells in WT recipients after LCMV infection^57^. However, our observation that IL-21:IL-21R signalling had a cell intrinsic role in Tfr cells suggests that IL-6 does not fully compensate for IL-21 in this setting. The explanation for this finding remains unknown, but may reflect the kinetics and localization of IL-21 production during Tfr cell differentiation and that IL-21 provides multiple inputs, including the upregulation of Bcl-6 (refs 48, 58, 59), inhibition of CD25 expression and destabilization of Foxp3 (refs 27, 28).

The relative percentages of Tfh cells in WT and *Il21r*^*−/−*^ mice reported by our group and others have varied^11^,^60^,^61^, influenced by the redundant roles of IL-21 and IL-6 in Tfh cell differentiation^40^,^41^. As shown in this study, a contributing factor to the variations in Tfh cell frequencies in *Il21r*^*−/−*^ mice may be *Il21r*^*−/−*^ Tfr cells that constitute a greater proportion of the total CXCR5^hi^PD-1^hi^ T follicular population than WT Tfr cells. Although the percentages of *Il21r*^*−/−*^ and WT Tfh cells were similar, the phenotype of Tfh cells was altered by *Il21r* deficiency with a notable increase in CXCR4 expression. CXCR4 is an α-chemokine receptor specific for stromal-derived-factor-1 (CXCL12), which is important in haematopoietic stem cell homing to the BM^62^ and for sorting of centroblasts into the GC dark zone^45^. A previous study has shown that CXCR4^+^CXCR5^+^ double-positive Tfh cells arise following influenza infection and could be identified within the GC^43^. CXCR4^+^CXCR5^+^ and CXCR5 single-positive Tfh cells both provided help for B-cell antibody production *in vitro*, but CXCR5 single-positive Tfh cells were superior at inducing B-cell proliferation^43^. Within the T-helper populations, IL-21R deficiency resulted in an expansion of CXCR4^+^ (CXCR5^−^) extrafollicular helper T cells. *Il21r*^*−/−*^ mice harbour increased levels of IgE^63^, but whether the expanded CXCR4^+^ extrafollicular subset contributes to this effect remains unknown.

The increased ratio of *Il21r*^*−/−*^ Tfr:Tfh cells reinforces the importance of distinguishing FoxP3^−^ follicular helper and FoxP3^+^ follicular Treg cell populations and may contribute to the important role of IL-21 acting on T cells for humoral immunity^64^,^65^. In this context, we provided evidence that the increased expansion or differentiation of *Il21r*^*−/−*^ Tfr cells has functional significance and was associated with a reduction in Tfh cells, IgG1^+^ GC B cells and antigen specific IgG1^+^ B cells and this finding is in line with previous observations on the influence of adoptively transferred functionally deficient Tfr cells^21^. The presence or absence of IL-21R on donor Treg cells had little effect on the total GC B cell population and, whilst we observed slightly reduced antigen specific B cells in FoxP3^DTR^ recipients of *Il21r*^*−/−*^ Treg cells, the greatest effect was on the broader IgG1^+^ population, which is consistent with Tfr cells acting to reduce the outgrowth of antigen non-specific B-cell clones^20^.

The propensity for *Il21r*^*−/−*^ T cells to differentiate into Tfr cells reflects the balance between responsiveness to IL-2 and IL-21 and reinforces the important role for IL-21 in humoral immunity. The containment of Tfr cells is important for a productive GC reaction and humoral immune response. Cytokines such as IL-21 that upregulate Bcl-6 have the capacity to reinforce antibody responses within the GC by constraining CD25 expression and the subsequent expansion of Tfr cells.

## Methods

### Patients

Peripheral blood samples of patients, their unaffected first-degree relatives and healthy volunteers were obtained upon written (parental) consent. The study protocols were approved by the National Institute of Allergy and Infectious Diseases Intramural Institutional Review Board in Bethesda, MD, the Sydney Children's Hospital Ethics committee and approved according to the Declaration of Helsinki by the local review board and Ministry of Health (0306-10-HMO).

### Mice

*Il21r*^*−/−*^ mice (created on 129 background and backcrossed onto C57BL/6ByJ to N12) were kindly provided by Dr Warren Leonard (NIH) and Ly5.1 C57BL/6 congenic mice were purchased from the Animal Research Centre in Perth, Australia. B6.129(Cg)-*Foxp3*^*tm3(DTR/GFP)Ayr*^/J (Foxp3^DTR^) mice were kindly provided by Alexander Rudensky^65^. The Garvan Institute and St Vincent's Hospital Animal Ethics Committee granted permission to perform animal experiments. Animals were housed under specific pathogen-free conditions and handled in accordance with the Australian code of practice for the care and use of animals for scientific purposes. Age-matched littermate mice used for experimental purposes were between 8 and 10 weeks of age. Conditional *Bcl6*^*−/−*^ mice on a mixed C57BL/6–129 Sv background in which CD4^+^ T cells were made genetically deficient in Bcl-6 (CD4Cre/WT:BCL6^fl/fl^) were described previously^66^.

### Immunizations

Mice were immunized (intravenous) with 2 × 10^8^ sheep red blood cells (IMVS, Australia) or (intraperitoneal, i.p.) with 100 μg of NP_13_-OVA absorbed to alum and spleens analysed at timepoints shown. For adoptive transfer experiments: Treg cells were enriched using the mouse CD4^+^ CD25^+^ Regulatory T Cell Isolation Kit, according to the manufacturer's instructions (Milentenyi Biotec, catalogue number 130-091-041). Body weight (50 μg kg^−1^) of diphtheria toxin was injected to deplete endogenous Treg cells on days −1 and 4 of immunization. Treg cells (5 × 10^5^) were transferred into FoxP3^DTR^ mice on day 0 of NP-OVA immunization. IL-2:IL-2mAb administration: in addition to SRBC immunization, IL-2:IL-2mAb complexes^47^,^67^ were injected i.p. every other day (days 0, 2, 4 and 6) with JES6-1 monoclonal antibody complexed with IL-2 and immunized i.p. with 2 × 10^8^ SRBC on day 0, splenocytes were analysed on day 7 via flow cytometry

### Bone marrow chimeras

Cohorts of B6 mice were lethally irradiated by a 137Cs source (B6: two doses of 0.45 Gy 4 h apart) and reconstituted the following day by intravenous injection with BM cells isolated from femurs and tibiae obtained by flushing bone with lymphocyte isolation media in sterile conditions. After irradiation, mice were maintained on antibiotic water containing cotrimoxazole (Roche) for 7 days. Irradiated CD45.1 B6 mice were reconstituted with 1 × 10^7^ BM cells from *Il21r*^*+/+*^ mice expressing the congenic markers CD45.2 and CD90.1 (Thy1.1) or CD90.2 (Thy1.2) to allow detection, combined with equal proportions BM from Thy1.2 CD45.2 WT or *Il21r*^*−/−*^ mice. Our reconstitution protocol typically resulted in an ∼40–60:60–40 ratio of WT:*Il21r*^*−/−*^ lymphocytes, and was contaminated with ∼10% of endogenous CD45.1^+^ radio-resistant T cells. Mice were used for immunization protocols as described above at 8 weeks post reconstitution.

### Immunofluorescence and FACS analyses

For flow cytometric analysis of human peripheral blood; cells were stained with antibodies directed against surface molecules and intracellular Foxp3. Antibodies purchased from BD Biosciences were: anti-CD3-BV786 (SK7, dilution 1/5), anti-CD25-PE (2A3, dilution 1/10), anti-CD127-BV421 (HIL-7R-M21, dilution 1/20), anti-CXCR5-A647 (RF8B2, dilution 1/30) and Strepdavidin-BV605 (dilution 1/100); antibodies purchased from eBioscience were anti-CD4-PE-Cy7 (RPA-T4, dilution 1/20) and anti-PD-1-biotin (eBioJ105, dilution 1/30). Nuclear Foxp3 was detected with anti-Foxp3-A488 (259D/C7, dilution 1/20) antibody using the eBioscience intracellular staining kit (catalogue number 00-5523-00) according to the manufacturer's instructions. For flow cytometric analysis of mouse lymphocytes; red blood cells were removed by hypotonic lysis. Fifty microlitres of a single cell suspension at 2 × 10^7^ cells per ml from the spleen and lymph nodes were stained in FACS buffer containing pre-titred antibodies in 96-well V-bottomed microtitre plates (Nunc, Roskilde, Denmark). Antibodies against surface molecules purchased from BD Bioscience were anti-CD4-PE-Cy7 (RM4–5, dilution 1/300), anti-CD25-APC & BV605 (PC61, dilution 1/100), anti-CD45.1-PE-Cy7 (A20, dilution 1/200), anti-CD45.2-APC-Cy7 (104, dilution 1/200), anti-CD90.1-eF450 (HIS51, dilution 1/200), anti-CD90.2-BV605 (53-2.1, dilution 1/200), anti-CXCR4-PE (2B11/CXCR4, dilution 1/50), anti-CXCR5-biotin (2G8, dilution 1/100), anti-GL7-PE (GL7, dilution 1/300), anti-FAS-FITC (Jo2, dilution 1/200), anti-IgG1-biotin (A85-1, dilution 1/100) and anti-Ki67-A647 (B56, dilution 1/100). Antibodies purchased from eBioscience were anti-CD19-eF450 (1D3, dilution 1/200), anti-ICOS-PE (C398.4A, dilution 1/300), anti-PD-1-FITC (RMP1–30, dilution 1/100), Streptavidin-PerCP-Cy5.5 and APC (dilution 1/300), anti-TCRβ-APC, FITC and PerCP-Cy5.5 (H57–597, dilution 1/300). For intracellular immunostaining of CXCR5^+^ cells, FoxP3 was detected with the anti-Foxp3-eF450 (FJK-16s, dilution 1/100) antibody purchased from eBioscience, whereas Bcl-6 was detected using the anti-Bcl-6-PE (112-91, dilution 1/50) antibody purchased from BD Bioscience using the BD intracellular staining kit (catalogue number 554714) according to the manufacturer's instructions. Detection of NP specific B cells was carried out by NIP-PE staining (dilution 1/100), to this end NIP-OSu (Biosearch Technologies) was conjugated to phycoerythrin (PE) (Molecular Probes)^68^. In short, PE was dialysed against 3% NaHCO_3_ over night at 4 °C, NIP-OSu was diluted in dimethylformamide and added to PE at a molar ratio of 20:1. The reaction mixture was rotated for 4 h at room temperature and afterwards dialysed against PBS overnight at 4 °C to remove unconjugated NIP. Intracellular P-STAT5 was detected with anti-P-STAT5-PerCP-Cy5.5 (47, dilution 1/5) antibody using the BD Phosphlow buffer III (Cat#558050) according to manufacturer's instructions. Cells were acquired using either a FACS Canto II or a LSRII SORP cytometer (BD Biosciences, CA) and analysed using Flowjo (Treestar, CA).

### Immunohistochemistry

Five-micrometre frozen OCT (Tissue Tek, Australia) spleen sections were fixed in ice-cold acetone for 8 min, washed and incubated with blocking reagent (Avidin/biotin (Dako)) at room temperature. Primary biotin or fluorochrome-conjugated antibodies were incubated at room temperature overnight. Antibodies against surface molecules purchased from BD Biosciences were anti-CD4-A488 (RM4–5, dilution 1/100) and anti-CD90.2-A647 (53-2.1, dilution 1/100). CD90.1 was detected using anti-CD90.1-eF450 (HIS51, dilution 1/100) antibody purchased from eBioscience, IgD was detected using anti-IgD-A488 & A647 (11-26c2a, dilution 1/100) antibodies purchased from Biolegend. Detection of FoxP3 was performed using TSA Kit 42, with HRP Streptavidin and Alexa Fluor 555 TyramideT (catalogue number Tx30955, Thermo Fisher) and the anti-FoxP3-biotin (FJK-16s, dilution 1/100) antibody purchased from eBioscience. Sections were analysed using a Leica DM I6000 SP8 confocal microscope (Leica Microsystems, Wetzlar, Germany). The images were processed using the Leica acquisition and analysis software ImageJ (Freeware NIH, Bethesda, USA) or Adobe Photoshop, version 7 (San José, CA).

### *In vitro* stimulations

CD4^+^ T cells were magnetic activated cell sorting (MACS) enriched from the spleens of WT mice and triplicate samples of 2 × 10^6^ cells per ml were preincubated for 5 min with or without 5 μg ml^−1^ IL-21RFc and then cultured for 3 days with 0.5 μg ml^−1^ soluble anti-CD3 (clone 145-2C11) and anti-CD28 (clone 37.51) monoclonal antibody titrated from a starting concentration of 1 μg ml^−1^. IL-21R/Fc chimera used to neutralize IL-21 *in vitro* was generated in house^69^. In brief, the DNA encoding the predicted extracellular domain (aa 1–235) of mouse IL-21R with a GSGS linker were amplified by PCR, and linked to mIgG2a Fc. The Fc domain contains four amino acid mutations (L285E, E368A, K370A and K372A) to minimize Fc binding and complement fixation. The resulting construct was subcloned into pEE12.4 (Lonza), a mammalian glutamine synthase expression vector, transfected into Chinese hamster ovary-K1SV cells, and grown in the presence of 25 μmol l^−1^ methionine sulphoximine. Following protein A purification, purity of IL-21R/Fc was checked by SDS–PAGE (Coomassie blue staining and silver stain, Lonza) and tested for presence of endotoxin (Lonza). *In vitro* BAF-3 proliferation assay measuring biological activity of IL-21R/Fc construct was conducted. For *in vitro* experiments using Bcl-6-deficient CD4^+^ T cells; CD4^+^ T cells were MACS enriched from the spleen of WT (WT/WT:BCL6^fl/fl^) and *Bcl6* conditional knockout mice (CD4Cre/WT:BCL6^fl/fl^) mice and triplicate samples of 2 × 10^6^ cells per ml were preincubated for 5 min with or without 5 μg ml^−1^ IL-21RFc and then cultured for 3 days with 0.5 μg ml^−1^ soluble anti-CD3 monoclonal antibody with or without 0.031 μg ml^−1^ anti-CD28 monoclonal antibody in the presence or absence of rmIL-21 (RnD Systems or made in house^70^). Following culture, live cells were immunostained and analysed by flow cytometry.

### Treg cell conversion *in vitro*

CD4^+^ cells were isolated from splenocytes by positive selection using magnetic beads. The CD4-negative fractions were irradiated with 3,000 rad and used as antigen presenting cells. To induce Treg cell conversion, CD4^+^ T cells were activated in the presence of 3 ng ml^−1^ recombinant human transforming growth factor-β1 with 1 μg ml^−1^ anti-CD3 monoclonal antibody (clone 145-2C11), either soluble in the presence of antigen presenting cells, or plate-bound together with soluble 2 μg ml^−1^ anti-CD28 monoclonal antibody (clone 37.51). Conversion efficiency was assessed by flow cytometry on day 3 after surface staining for CD4 and 7-aminoactinomycin D (7-AAD) followed by intracellular staining for Foxp3.

### Statistics

*P*-values between data sets were determined by two-tailed Student's *t*-test, assuming equal variance or one-way analysis of variance where indicated. Mixed BM chimeras were analysed in individual mice by pairwise analyses (*t*-test).

### Data availability

The authors declare that the data supporting the findings of this study are available within the article and its Supplementary Information files.

## Additional information

**How to cite this article:** Jandl, C. *et al*. IL-21 restricts T follicular regulatory T cell proliferation through Bcl-6 mediated inhibition of responsiveness to IL-2. *Nat. Commun.* **8,** 14647 doi: 10.1038/ncomms14647 (2017).

**Publisher's note:** Springer Nature remains neutral with regard to jurisdictional claims in published maps and institutional affiliations.

## Supplementary Material

Supplementary Information Supplementary Figures
