# Supplementary material for: IL-21 restricts T follicular regulatory T cell proliferation through Bcl-6 mediated inhibition of responsiveness to IL-2
Source: Nat Commun. 2017 Mar 17;8:14647. doi: 10.1038/ncomms14647 (PMC5357862; doi:10.1038/ncomms14647)
Supplement: Supplementary Information — Supplementary Figures [file ncomms14647-s1.pdf]

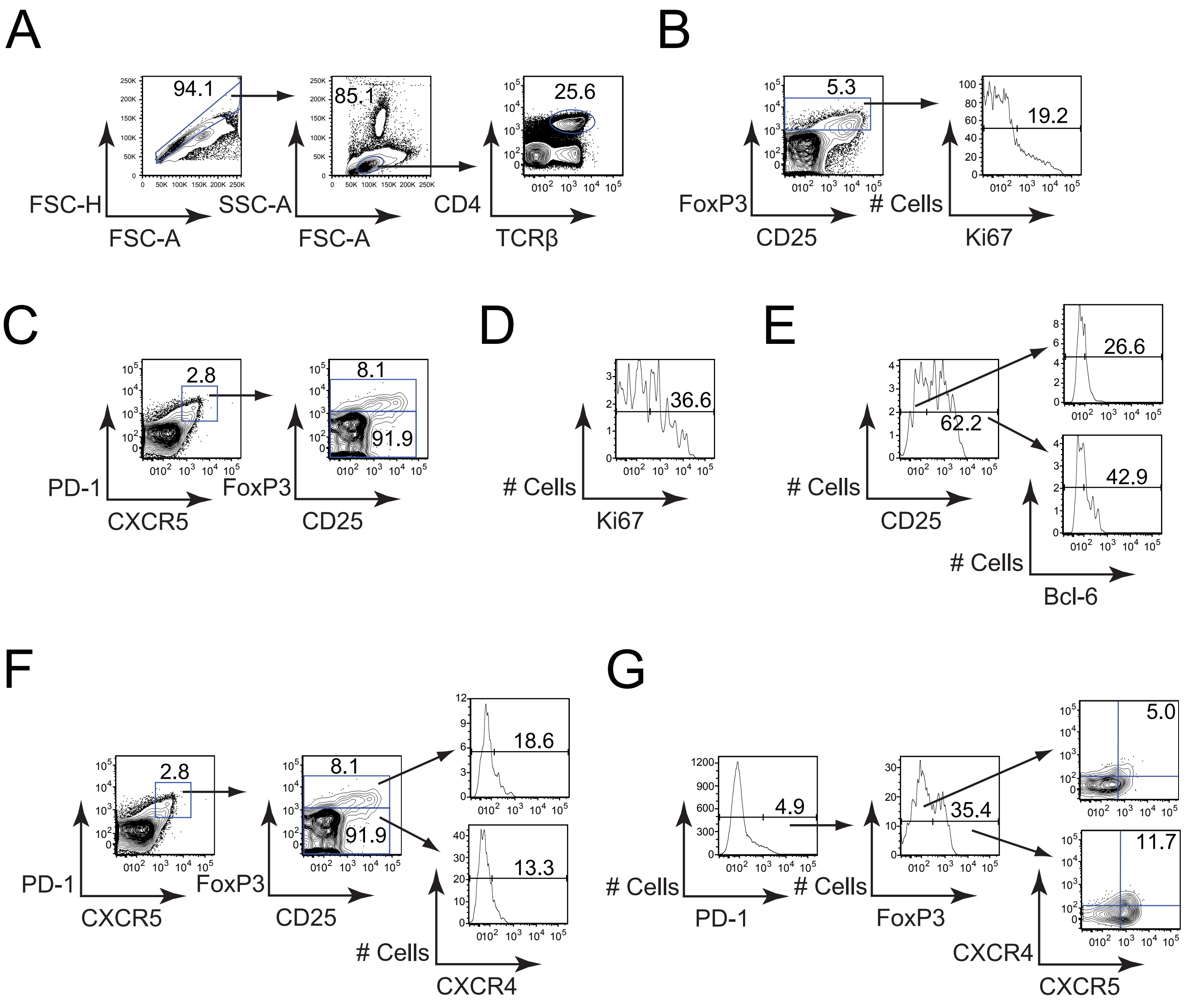

Supplementary Figure 1.

Gating strategy to differentiate CD4+ T cell subsets in immunized mice.

Doublets were excluded using appropriate FSC-H/FSC-A gates, followed by gating on lymphocytes using appropriate SSC-A/FSC-A gates and CD4+ T cells were selected as TCRβ+ CD4+ cells (a). Regulatory T cells were defined as CD4+ T cells that were FoxP3+ and proliferating Tregs were selected as Ki67+ FoxP3+ CD4+ T cells (b). To select Tfh/Tfr cells, the CXCR5+ PD-1+ CD4+ T cell population was separated into FoxP3+ Tfr cells and FoxP3- Tfh cells (c). Proliferating Tfr cells were defined as Ki67+ FoxP3+ Tfr cells (d). FoxP3+ Tfr cells were differentiated into CD25+ and CD25- subsets and Bcl-6 expression was assessed on both populations (e). CXCR4 expression was assessed on both FoxP3+ Tfr cells and FoxP3- Tfh cells (f). To identify T extrafollicular helper cell subsets PD-1high CD4+ T cells were differentiated based upon their FoxP3 expression to gate on FoxP3- CXCR5- CXCR4+ T extrafollicular helper cells and FoxP3+ CXCR5- CXCR4+ T extrafollicular regulatory cells (g). Data shown are from one mouse and are representative of five independent experiments.

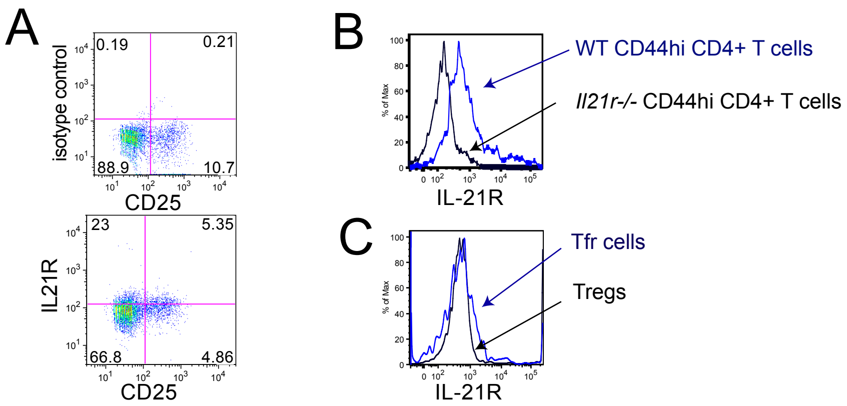

Supplementary Figure 2.

Representative FACS dot plots showing (a) IL-21R expression relative to CD25 expression on CD4+ T cells, representative FACS histogram overlays showing (b) the expression of IL-21R on CD44hi CD4+ T cells from Il21r-/- and WT mice and (c) CD25+ Tregs cells versus CD25+ Tfr cells.

Data are representative of 2 experiments, where n=5.

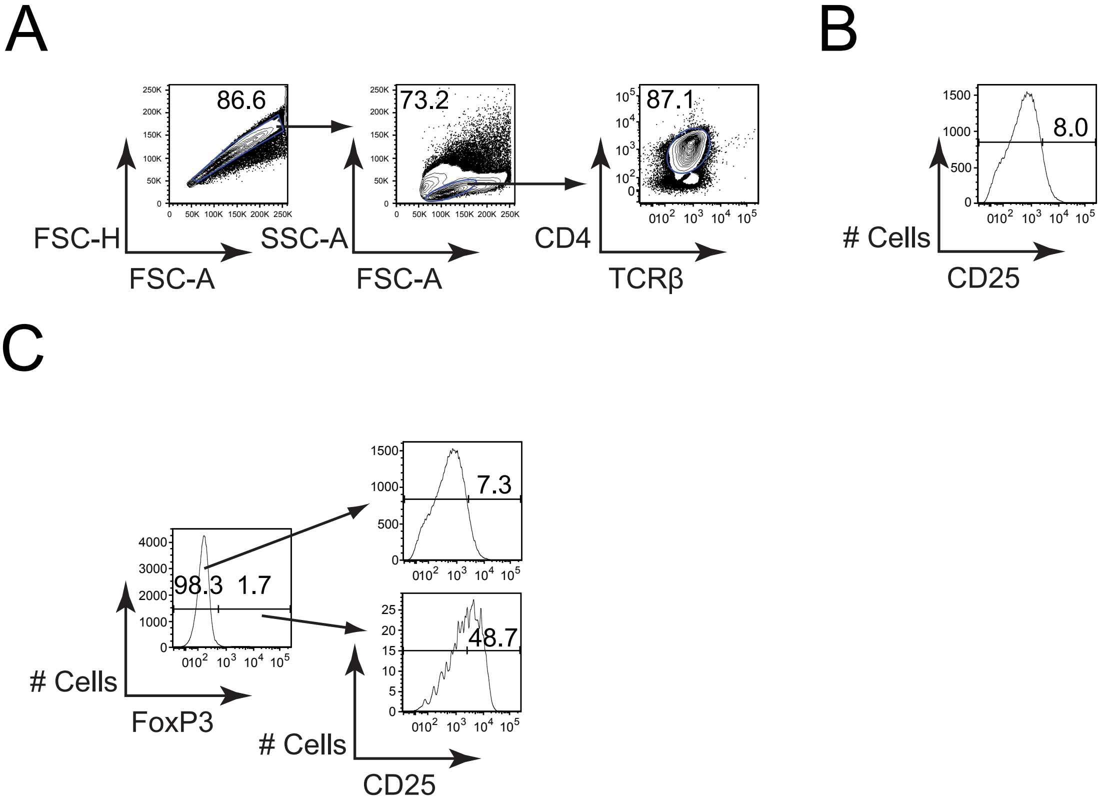

Supplementary Figure 3.
Gating strategy to select murine CD4+ T cell populations after 3 days of *in vitro* cuture.
Doublets were excluded using appropriate FSC-H/FSC-A gates, followed by gating on lymphocytes using appropriate SSC-A/FSC-A gates and CD4+ T cells were identified as TCRβ+ CD4+ cells (a). CD4+ T cells expressing high levels of were identified as CD25hi CD4+ TCRβ+ cells (b). The CD4+ T cell population was divided into FoxP3+ and FoxP3- populations, and CD25hi cells were identified in both populations (c). Data shown are from one mouse and are representative of two independent experiments.

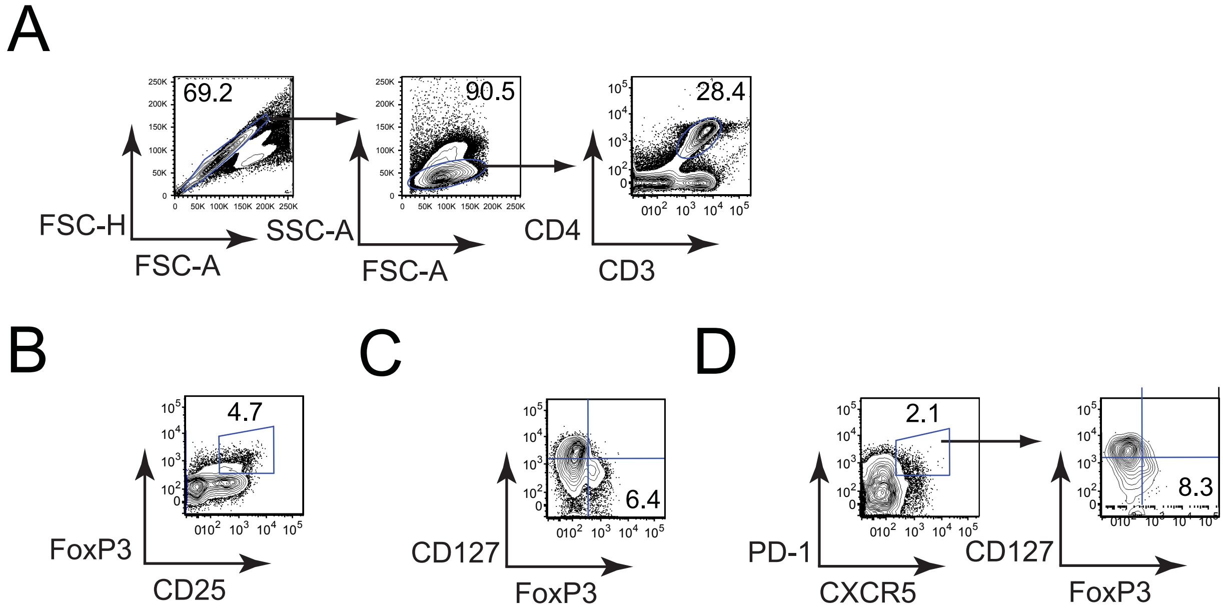

Supplementary Figure 4.

Gating strategy to differentiate human CD4+ T cell subsets.

Doublets were excluded using appropriate FSC-H/FSC-A gates, followed by gating on lymphocytes using appropriate SSC-A/FSC-A gates and CD4+ T cells were selected as CD3+ CD4+ cells (a). Regulatory T cells were defined either as CD4+ T cells that were FoxP3+ CD25+ (b) or as CD4+ T cells that were FoxP3+ CD127low (c). To select Tfh/Tfr cells the CXCR5+ PD-1+ CD4+ T cell population was differentiated into FoxP3+ Tfr cells and FoxP3- Tfh cells (d). Data shown are from one patient and are representative of four independent experiments.

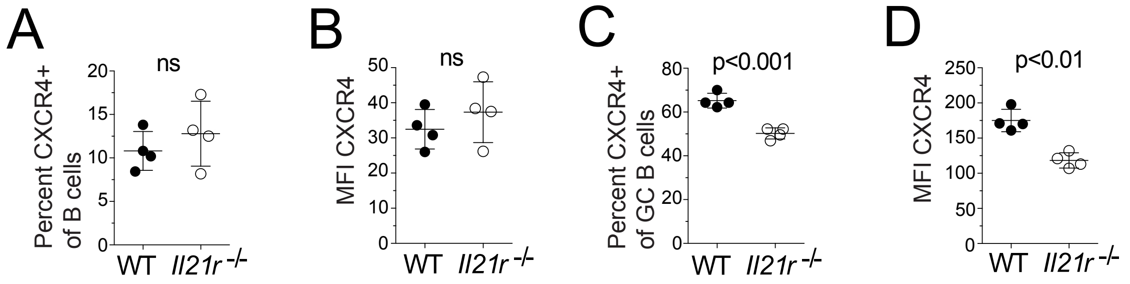

Supplementary Figure 5.
Analyses of Germinal Centre B cells from Figure 3 showing (a) the percentage of CXCR4 expressing Germinal Centre B cells and (b) the mean fluorescence intensity (MFI) of CXCR4 expression on total B cells (c) the percentage of CXCR4 expressing Fas+GL7+ germinal center B cells and (d) the mean fluorescence intensity (MFI) of CXCR4 expression on Fas+GL7+ germinal center B cells.

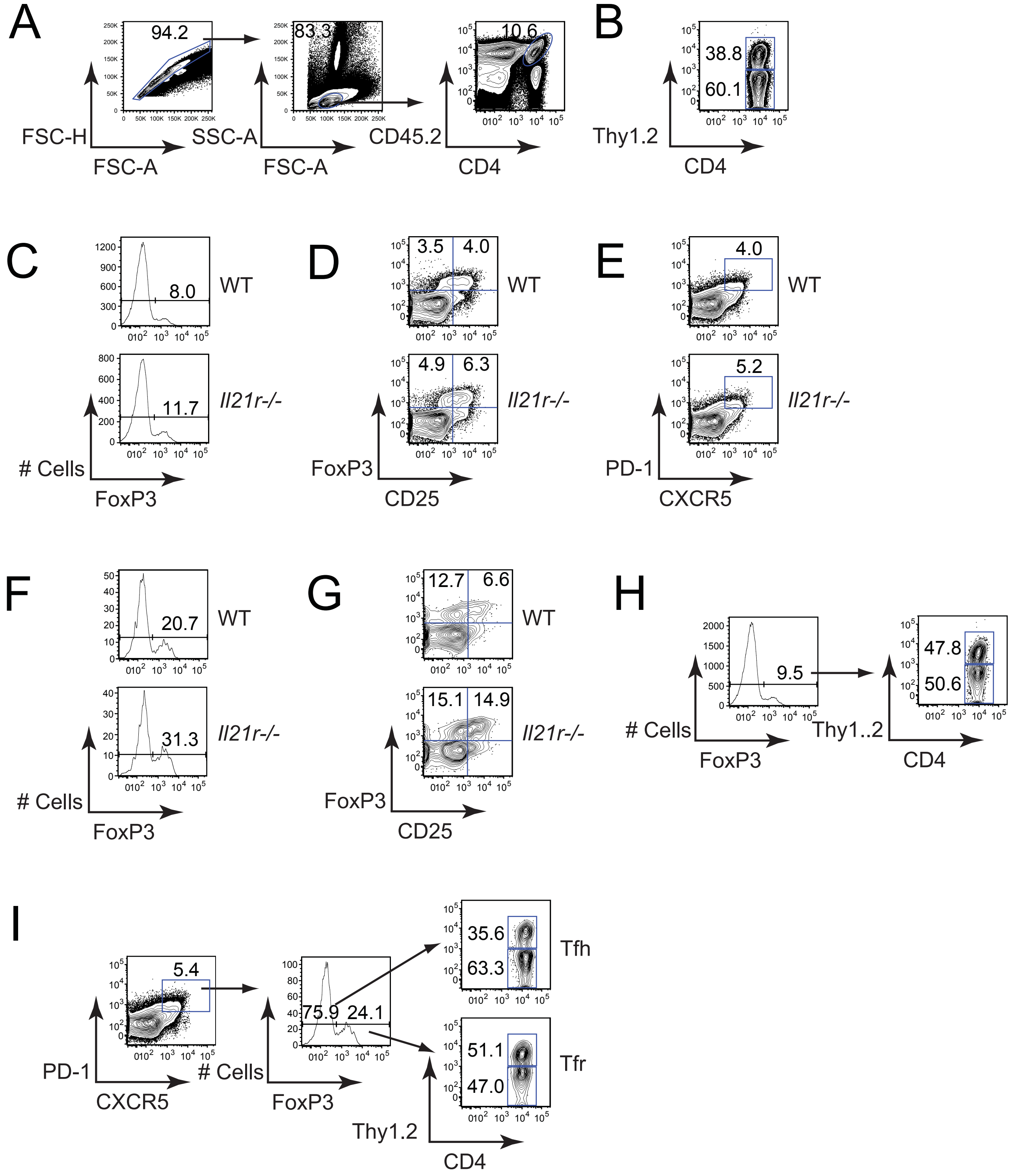

Supplementary Figure 6.
Gating strategy to differentiate Thy1.1+ WT and Thy1.2+ *Il21r-/-* donor CD45.2 CD4+ T cell subsets in CD45.1+ recipient mice. Doublets were excluded using appropriate FSC-H/FSC-A gates, followed by gating on lymphocytes using appropriate SSC-A/FSC-A gates and donor CD4+ T cells were selected as CD45.2+ CD4+ cells (a). CD45.2+ donor CD4+ T cells were separated into Thy1.2- WT cells (Thy1.1+) and Thy1.2+ *Il21r-/-* cells (b). Treg cells were identified by gating on FoxP3+ Thy1.1+ WT and Thy1.2+ *Il21r-/-* CD4+ T cells (c). FoxP3 expression on CD25+ and CD25- Thy1.1+ WT CD4+ T cells and Thy1.2+ *Il21r-/-* CD4+ T cells was assessed (d). Follicular CD4+ T cell subsets were selected by gating on CXCR5+ PD-1+ WT and *Il21r-/-* donor CD4+ T cell subsets (e). Follicular CD4+ T cells were separated into FoxP+ Tfr cells and FoxP3- Tfh cells in both WT and *Il21r-/-* donor CD4+ T cell populations (f). FoxP3 expression on CD25+ and CD25- follicular CD4+ T cells in both WT and *Il21r-/-* donor populations was assessed (g). Donor regulatory T cells were identified by gating on FoxP3+ CD45.2+ donor CD4+ T cells and were subsequently separated into Thy1.2- WT (Thy1.1+) cells and Thy1.2+ *Il21r-/-* cells (h). Donor follicular cell populations were identified by gating on CXCR5+ PD-1+ CD45.2+ donor CD4+ T cells, which were then differentiated into FoxP3- Tfh cells and FoxP3+ Tfr cells. Then the contribution of Thy1.2- WT (Thy1.1+) cells, and Thy1.2+ *Il21r-/-* cells to these populations was assessed (i). Data shown are from one mouse and are representative of two independent experiments.

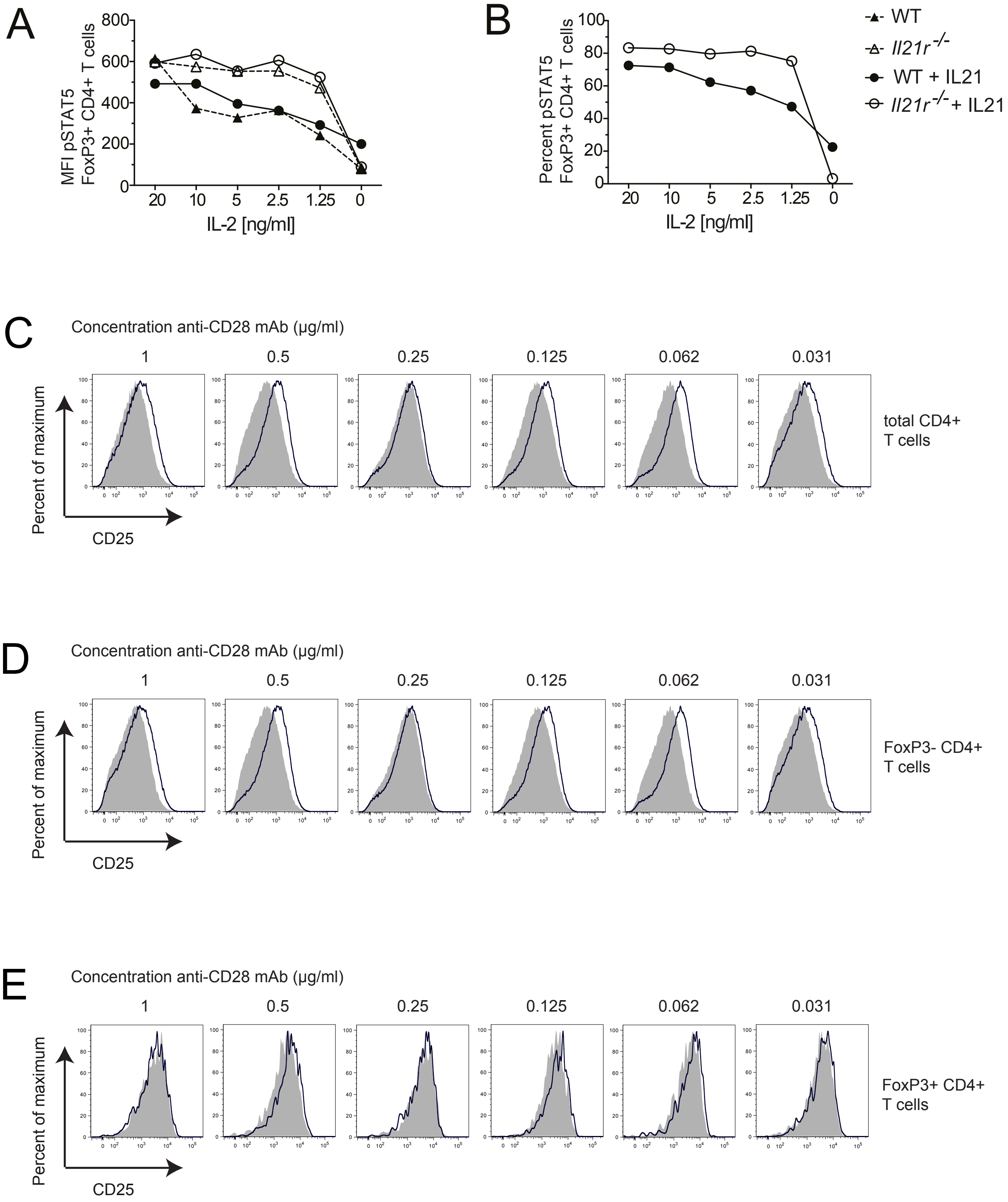

Supplementary Figure 7.

Il21r-/- Tregs have increased STAT5 activation in response to IL-2. Splenocytes from Il21r-/- and WT mice were treated ex vivo for 15min with indicated concentrations of rmIL-2 +/- rmIL-21. Intracellular phosphorylated P-STAT5 expression within CD25+Foxp3+CD4+TCRb+ T cells shown as (a) mean fluorescence intensity (MFI) P-STAT5 and (b) P-STAT5+ cells as a percentage of CD25+FoxP+CD4+TCRb+ T cells and is representative of two experiments with similar results.

CD4+ T cells from the spleen of C57BL/6 mice were cultured ex vivo in the presence of anti-CD3 mAb and decreasing concentrations of anti-CD28 mAb, in the presence or absence of IL-21 neutralization (IL-21RFc) for 3 days. Representative FACS histogram overlays from Figure 6 showing the immunofluorescence intensity (MFI) of the expression of CD25 on (c) TCRb+CD4+ T cells (d) Foxp3- TCRb+CD4+ T cells and (e) Foxp3+ TCRb+CD4+ T cells in the absence (filled histograms) or presence (black lines) of 5µg/ml of IL-21RFc.

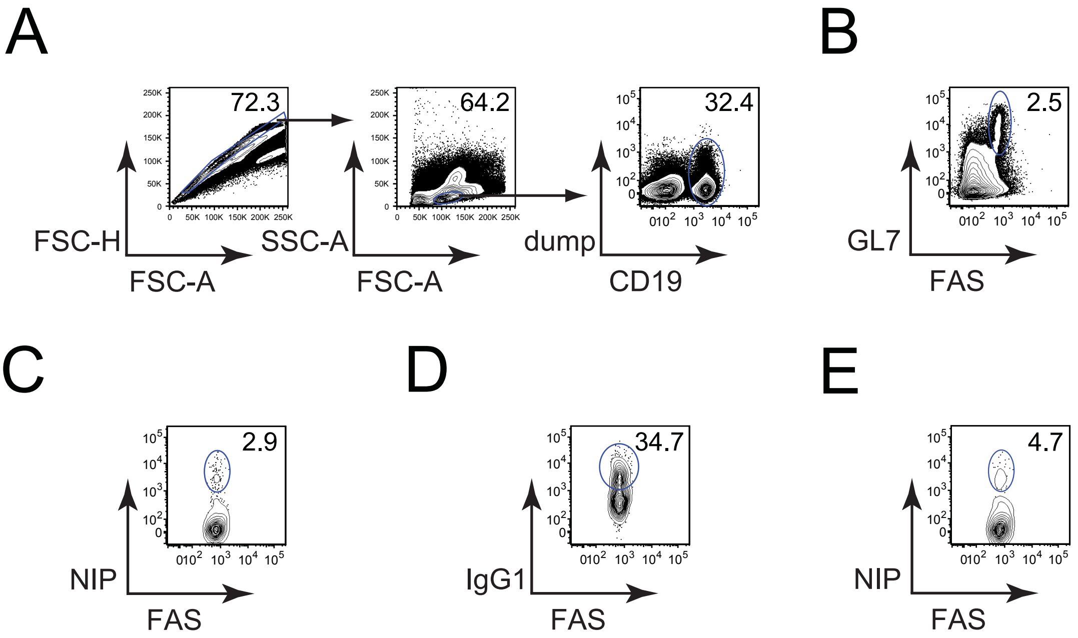

Supplementary Figure 8.

Gating strategy to select Germinal Centre B cell populations in mice after immunization.
Doublets were excluded using appropriate FSC-H/FSC-A gates, followed by gating on lymphocytes using appropriate SSC-A/FSC-A gates and B cells were identified as CD19+ lymphocytes (a). GC B cells were selected by gating on GL7+ FAS+ B cells (b). NP-specific GC B cells were selected by gating on NIP+ GC B cells (c). IgG1 producing GC B cells were selected by gating on IgG1+ FAS+ GC B cells (d). NP-specific IgG1+ producing GC B cells were selected by gating on NIP+ FAS+ IgG1+ GC B cells (e). Data shown are from one mouse and are representative of two independent experiments.
